# Composition and Use of Cannabis Extracts for Childhood Epilepsy in the Australian Community

**DOI:** 10.1038/s41598-018-28127-0

**Published:** 2018-07-05

**Authors:** A. Suraev, N. Lintzeris, J. Stuart, R. C. Kevin, R. Blackburn, E. Richards, J. C. Arnold, C. Ireland, L. Todd, D. J. Allsop, I. S. McGregor

**Affiliations:** 1 The Lambert Initiative for Cannabinoid Therapeutics, School of Psychology, The University of Sydney, Sydney, 2050 Australia; 2 Addiction Medicine, Central Clinical School, Faculty of Medicine, The University of Sydney, Sydney, 2006 Australia; 3 The Langton Centre, Drug and Alcohol Services, South East Sydney Local Health District, NSW Health, Surry Hills, 2010 Australia; 4 Department of Pharmacology, Faculty of Medicine, University of Sydney, Sydney, NSW 2006 Australia; 5 Epilepsy Action Australia, Sydney, Australia

## Abstract

Recent surveys suggest that many parents are using illicit cannabis extracts in the hope of managing seizures in their children with epilepsy. In the current Australian study we conducted semi-structured interviews with families of children with diverse forms of epilepsy to explore their attitudes towards and experiences with using cannabis extracts. This included current or previous users of cannabis extracts to treat their child’s seizures (n = 41 families), and families who had never used (n = 24 families). For those using cannabis, extracts were analysed for cannabinoid content, with specific comparison of samples rated by families as “effective” versus those rated “ineffective”. Results showed that children given cannabis extracts tended to have more severe epilepsy historically and had trialled more anticonvulsants than those who had never received cannabis extracts. There was high variability in the cannabinoid content and profile of cannabis extracts rated as “effective”, with no clear differences between extracts perceived as “effective” and “ineffective”. Contrary to family’s expectations, most samples contained low concentrations of cannabidiol, while Δ^9^-tetrahydrocannabinol was present in nearly every sample. These findings highlight profound variation in the illicit cannabis extracts being currently used in Australia and warrant further investigations into the therapeutic value of cannabinoids in epilepsy.

## Introduction

Severe epilepsies of infancy and early childhood are chronic conditions often characterized by recurrent, unprovoked seizures and developmental delay^1^. Treatment-resistant epilepsy affects 20–30% of patients^2,3^ despite increasing availability of novel antiepileptic drugs with new modes of action, fewer side-effects, and improved tolerability profiles. Treatment-resistance is defined as a failure of two appropriate trials of antiepileptic drugs (alone or in combination) to achieve seizure-freedom^2^, with the chances of efficacy diminishing with each new antiepileptic introduced^4^. Some treatment-resistant patients may undergo surgical intervention to reduce seizures, while others may be treated with multiple antiepileptic drugs with the aim of achieving therapeutic ‘synergism’, despite limited evidence for this approach^5,6^.

Recurrent seizures are only one manifestation of treatment-resistant epilepsy, which is a multifactorial condition^7^. Additionally, normal developmental trajectories are disrupted, with increased risk of intellectual disability and behavioural and psychosocial dysfunction^7–9^. Given such a troubling prognosis, parents of afflicted children are often prepared to experiment with alternative therapeutic interventions. An example of this is the use of artisanal unregistered cannabis products for childhood epilepsy, a phenomenon increasingly widespread in both Australia and North America^10–13^. This has attracted considerable media attention^14,15^, which in turn, has strongly propelled community interest, uptake, and advocacy of cannabinoid use for the treatment of epilepsy around the world.

The phenomenon is not without supporting scientific evidence. Many preclinical studies have identified potent anticonvulsant effects of various cannabinoids in animal models of epilepsy^16–19^, and a mechanistic understanding of such effects is emerging^20,21^. Translation of these preclinical results into clinical outcomes is developing, with a randomised controlled trial showing therapeutic effects of cannabidiol (CBD) in Dravet syndrome, a severe form of childhood epilepsy^22^. When CBD was used as an adjunct to standard antiepileptic drug treatment, 43% of patients achieved at least a 50% reduction in seizures compared to 27% with placebo^22^. Similar positive results were also identified in a more recent randomised controlled trial of CBD in children with Lennox-Gastaut syndrome^23^. Other supporting evidence comes from an open-label study^24^, retrospective cohort studies^12,25^, observational surveys^10,11,26^, and case series and reports^27–31^, all highlighting the anticonvulsant potential of cannabis extracts in childhood epilepsy.

With CBD, some uncertainty surrounds the role of pharmacokinetic interactions in the observed seizure reduction in Dravet syndrome^32^. Recent pharmacokinetic analyses indicate that co-administered CBD causes potentially clinically significant changes in plasma levels of clobazam and its primary metabolite, *N*-desmethylclobazam, in patients via CBD inhibition of specific cytochrome P450 enzymes^32,33^. The relevance of these and other^34^ pharmacokinetic interactions to cannabinoid therapeutic effects requires further elucidation as does examination of the factors that determine why some patients with epilepsy respond better to cannabinoids than others.

In countries such as Australia where legal cannabis-based products are still highly restricted, some parents are sourcing illicit cannabis extracts for their children. A recent Australian nationwide survey of 976 people with epilepsy indicated that 13% of parents had tried or were using illicit cannabis extracts to manage their child’s seizures^13^. The main reasons for this were to manage treatment-resistant seizures and to reduce the antiepileptic drug side-effects that their child was experiencing. However, illicit cannabis products are typically of unknown composition, quality and safety, and are unlikely to be optimised for anticonvulsant efficacy. Moreover, 40% of parents in that survey identified possible legal ramifications as a key concern in using illicit cannabis products.

The primary aim of the present study was to explore Australian family’s experiences with, and perspectives on, cannabis extract use for childhood epilepsy. A semi-structured interview asked detailed questions about the child’s epilepsy and past treatment history, the perceived effectiveness of cannabis products, the ease of access and reliability of access for such products, and potential concerns of using an illicit substance including possible legal ramifications. In addition to interviewing families who had used such products, we interviewed families who had never used them. This allowed us to investigate the factors that may be important in driving the decision of parents to administer cannabis extracts to a child with epilepsy.

Australian street cannabis tends to have high Δ^9^-tetrahydrocannabinol (THC) levels but very low and often undetectable levels of CBD^35^. This led us to hypothesize that the illicit extracts being used by Australian families might be THC-rich and CBD-poor. This is of interest given the pre-eminence of CBD products in current clinical trials for epilepsy. Preclinical studies have demonstrated anticonvulsant effects of other plant-derived cannabinoids including cannabidivarin (CBDV)^16,18,36^, tetrahydrocannabivarin (THCV)^17^, and cannabidiolic acid (CBDA)^19^ in addition to terpenoids from the cannabis plant such as β-caryophyllene^37^. Some preclinical studies have also identified anticonvulsant effects of THC^38–40^, while others have identified proconvulsant effects at higher doses^41–43^. This raises the intriguing possibility that any anticonvulsant effects of illicit cannabis extracts may be somewhat independent of CBD. Accordingly, a secondary aim of the study was to examine the composition of such extracts and specifically compare those perceived as “effective” by families, with those perceived as “ineffective”.

## Methods

The study involved semi-structured interviews with families of children with epilepsy, and where possible, collection and subsequent laboratory analysis of samples of cannabis extracts used by participating families.

### Participants and semi-structured interview

We conducted face-to-face, semi–structured interviews with families who had a child aged 16 years or under with a diagnosis of epilepsy. These included families who reported using cannabis extracts as a treatment for their child’s epilepsy (who were either currently using cannabis extracts or had previously used but had now stopped) and families who reported having never tried cannabis for their child’s epilepsy. As this was an exploratory study, we aimed to recruit 50 participants to adequately saturate the data pertaining to their experiences. The age range of 16 years or under was chosen to avoid any burden on the child by only seeking consent, and only interviewing, the parent or guardian on their experiences and perspectives. This also avoided some potentially ethically sensitive issues regarding interviewing children about their parent’s choice of treatments.

The interview was delivered by a female psychologist (A.S. or R.B.) to one or both parents/guardians.  Interviews were conducted either at the participant’s home, or at a private clinic room at the Brain and Mind Centre, Sydney or the Centre for Children’s Health Research, Brisbane, and lasted approximately 1.5 hours. All study data were collected during the single visit. Participants were recruited between June 2016 – December 2017 through social and conventional media, non-profit advocacy groups, community health clinics and centres, and snowball sampling. All research was performed in accordance with relevant guidelines and regulations, with approval obtained from University of Sydney Human Research Ethics Committee (2015/234) and Children’s Health Queensland Hospital and Health Service Human Research Ethics Committee (HREC/16/QRCH/267). All participants provided written informed consent prior to interview. All families were compensated for their time with a $50 gift voucher.

De-identified study data were collected on paper and managed using REDCap electronic data capture tools hosted at The University of Sydney^44^. If more than one primary caregiver was present at interview, their responses were merged as one on the interview form. The interview schedule was designed to investigate family’s experiences with and opinions on cannabis extracts as a treatment for their child’s epilepsy (full list of survey questions available in Supplementary Methods S1). It comprised both structured and open-ended questions that allowed exploration of relevant issues, including questions regarding demographic and clinical data; child’s schooling; reason for using or not using cannabis; for those who tried cannabis to treat child’s epilepsy – questions about the use of cannabis extracts, such as duration of use, reliability of access, and cost; questions specific to each cannabis extract tried, including type of product, mode of use, and dosage; questions about perceived efficacy of cannabis extract on an adapted Patient Global Impression of Change (PGIC) scale^45^ (graded on a seven-point rating scale, from 1 (very much improved) to 7 (very much worse) with a score of 4 being no change) and a percentage rating of change in seizure frequency, as well as changes to anticonvulsant medication and effects on quality of life and behaviour; adverse effects; and questions on attitudes towards medicinal cannabis and preferences of use. The interview schedule was piloted and refined using two neurologists and five participants to ensure appropriate wording. Interview questions were purposefully chosen to enable harmonisation with a subsequent survey conducted by our group exploring medicinal cannabis use in Australia.

### Measures of child quality of life and behaviour

Quality of life in children was assessed via parent self-report using age-appropriate quality of life measures: TNO-AZL Preschool Children Quality of Life Questionnaire (TAPQOL) (1–5 years of age)^46,47^ and the shortened Quality of Life in Childhood Epilepsy (QOLCE-55) (6–16 years of age)^48,49^. The TAPQOL is a more generic measure of quality of life that has been previously administered in children with epilepsy in the younger age group^50,51^. Higher scores indicated better quality of life. Child behaviour problems were assessed in all children using an age-appropriate Child Behaviour Checklist (CBCL) used extensively in children with epilepsy^52^. CBCL scores are presented as T scores ranging from 0 to 100, for which scores equal to or less than 50 represent few or no behavioural problems, whereas the highest score 100 indicates extensive behavioural problems.

### Sample collection

A sample of the cannabis extract (2 mL) was collected at interview. Providing such a sample was entirely voluntary and did not form part of the inclusion criteria for participation in the study. These samples were analysed for cannabinoid and terpenoid content, as detailed below. To determine exact concentration, portable scales were used to determine weight of a single dose using the participant’s own dispensing instrument. Dosage (mg/kg/day) was calculated based on the concentration of the cannabis extract (mg/g), the amount administered by the family per day (e.g. millilitres, drops, or physical weight), and the child’s body weight as reported by the parent. Families were given the option to receive individualised feedback on the cannabinoid profile of their extract. All families were de-identified once this feedback was complete. To validate the family’s reported use of the cannabis extract for their child, all families were also asked to provide a sample of their child’s urine for urinalysis of cannabinoids and associated metabolites. Authority to possess scheduled substances for the purpose of this research was provided by the relevant state government Department of Health.

### Sample analysis

Extraction of phytocannabinoids from cannabis extracts was adapted from a validated method^53^. In brief, samples were diluted to 10 mg/mL with ethanol and spiked with diazepam (internal standard). The samples were then capped, roto-racked, winterized and centrifuged. After a 1/10 dilution in ethanol the process was repeated. Post-centrifuge, 200 µL of each sample was dried under nitrogen stream and reconstituted in 1 mL of 60:40 0.1% formic acid and acetonitrile for analysis via liquid chromatography-tandem mass spectrometry (LC-MS/MS), using a Shimadzu Nexera® ultra-high-performance liquid chromatograph coupled to a Shimadzu 8030 triple quadrupole mass spectrometer (Shimadzu Corp., Kyoto, Japan).

Similarly, terpenoids were extracted from cannabis extract samples (10 mg) with ethanol (2 mL). The ethanolic extracts were spiked with lauryl acetate (internal standard), then capped, vortexed, and centrifuged. Terpenoid quantification was performed via direct injection into a Shimadzu GC-2010 Plus GC system coupled to a Shimadzu QP-2010 MS. Presence of cannabinoids and terpenoids in the cannabis extract sample were determined using a cut-off of 1 ng/mg.

Phytocannabinoid urinalysis was performed as reported previously^54^ with minor modification, where urine samples (0.5 mL) were hydrolysed with red abalone β-glucuronidase and extracted using supported liquid extraction with methyl *tert*-butyl ether (5 mL). Extracts were dried under nitrogen, and reconstituted in 60:40 0.1% formic acid and acetonitrile (100 µL). Phytocannabinoids were then quantified via LC-MS/MS. Creatinine was also quantified to account for urinary dilution. Full methods can be found in the Supplementary Methods S2.

### Data analysis

Survey responses were exported from REDCap electronic data capture tool and tabulated in a spreadsheet. Analyses were conducted using IBM SPSS 24.0 (IBM Corp., Armonk, N.Y., USA) and graphs were created using GraphPad Prism 7 for Mac OS X (GraphPad Software, La Jolla, California, USA). Results were summarised using descriptive statistics (frequency, percentage of valid responses). Thematic analysis was utilised to categorise open-ended responses as previously described^55^. Responses were coded by two independent reviewers (A.S. and R.B.), before decisions concerning the salience of themes chosen for the current research and the allocation of responses under those themes were discussed and agreement was reached. Examples of participant’s responses for each theme are available in the Supplementary Methods S3. Due to the small number of families who had previously used cannabis extracts but had now stopped, data were merged to form two groups which included those who are currently or have previously used cannabis extracts (n = 41) and those who have never used cannabis extracts (n = 24) for their child’s epilepsy. Chi-square test of significance and Fisher’s exact test (two-sided) for categorical variables were used to analyse data collected at interview. The Wilcoxon Signed-ranks non-parametric test was used when comparing two related samples. Independent samples t-tests with Bonferroni correction were used to assess differences in child’s quality of life and behaviour between using and non-using families.

Some families co-administered more than one cannabis extract to their child: in this case, cannabinoid concentration data for each individual cannabis extract were merged prior to data analysis to provide a total daily dosage. An improvement in symptom control (“effective”) was defined as a 1–3 on the PGIC and/or ≥50% reduction in seizure frequency based on the family’s observation. A non-response or deterioration in symptoms (“ineffective”) was defined as a 4–7 on the PGIC and/or <50% reduction in seizure frequency based on the family’s observation. If the family were unsure of the effects the cannabis extract was having on their child’s epilepsy, such as when antiepileptic drug treatment was concurrently introduced or weaned down, the sample was excluded from the final analysis (n = 5).

The data for continuous variables were expressed as mean ± standard deviation. Independent samples t-tests were used to compare the average cannabinoid and terpenoid concentrations between samples perceived “effective” and “ineffective”. Levene’s test was used to examine homogeneity of variance. An alpha level of 0.05 was used for all statistical tests. To correct for multiple comparisons, the Benjamini-Hochberg correction was used to control the false discovery rate, with *q* values ≤ 0.05 considered significant^56^.

### Data Availability

The datasets generated during and/or analysed during the current study are available from the corresponding author on reasonable request.

## Results

### Demographics and child clinical history

**Figure 1:**
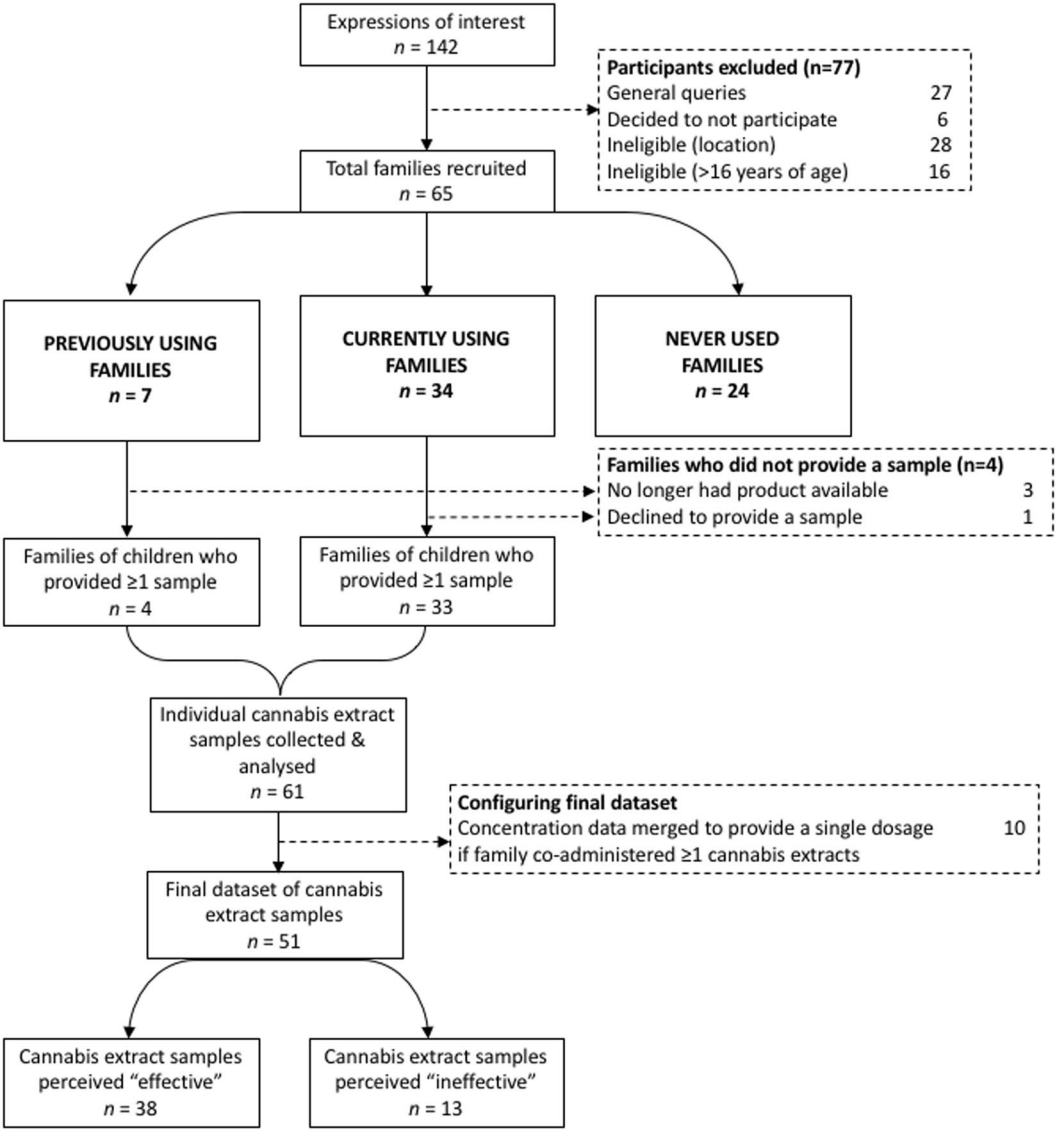
Flow diagram of study’s participant recruitment and collection of cannabis extract samples from families who were currently using or had previously used cannabis extracts to treat their child’s epilepsy.

Data were collected from interviewing 65 families of children with epilepsy. All of the interviewees were the biological parent(s) of the child with epilepsy. All participating families contained only one child with epilepsy. The participating families included 41 families who reported using cannabis extracts as a treatment for their child’s epilepsy (34 families who were currently using cannabis extracts and seven families who previously used but had now stopped), while 24 reported having never tried cannabis for their child’s epilepsy (see flow diagram in Fig. 1). The children included 37 females (57%) and 28 males (43%) with an average age of 8.8 ± 4.6 years (range 1–16) (Table 1). According to the family, 44 children (68%) had an epilepsy diagnosis of unknown aetiology (see Supplementary Table S1 for full list of epilepsy diagnoses), three children (4.6%) had structural-metabolic cause epilepsy, and 22 (34%) had a genetic cause. The average age of seizure onset was 3.3 ± 3.9 years (range 0–14.2). Seventeen children (26%) had undergone genetic testing and were positive for a mutation: six children (9.2%) tested positive for sodium voltage-gated channel alpha subunit 1 (SCN1A) mutation (all receiving cannabis extracts) while the remainder comprised individual cases of specific gene mutations (see Table 1 for full list). For the remaining children with epilepsy of genetic cause, four were presumed genetic (testing was inconclusive) and one family could not recall the positive gene name.

**Table 1:** Demographics and clinical history of children with epilepsy based on cannabis extract use history using valid percentage.

	Currently or have previously used	Never used	Total
	N (%)	N (%)	N (%)
Number of participants	41	24	65
Female	23 (56%)	14 (58%)	37 (57%)
Child’s schooling			
Mainstream	17 (42%)	10 (42%)	27 (42%)
Special education	16 (39%)	8 (33%)	24 (37%)
Unable to attend school	8 (19%)	6 (25%)	14 (22%)
Epilepsy aetiology			
Unknown	23 (56%)	21 (88%)	44 (68%)
Genetic	15 (37%)	7 (29%)	22 (34%)
Structural-metabolic	3 (7%)	—	3 (4%)
*Positive mutation	12 (29%)	5 (21%)	17 (26%)
SCN1A			6
DNM1			2
SCN2A			1
PCDH19			1
SCN9A			1
Trisomy 16			1
17p13.3 microdeletion			1
TSC2			1
Mutation on C5 and C17			1
C.316 C > T			1
KCNQ2			1
Drug-resistant epilepsy	35 (85%)	12 (50%)	47 (72%)
Status epilepticus episodes	31 (76%)	14 (58%)	45 (69%)
AEDs perceived efficacy			
<50% reduction	26 (63%)	5 (21%)	31 (48%)
≥50% reduction	15 (37%)	17 (71%)	32 (49%)
Never started	1 (2%)	1 (4%)	2 (3%)
	**Mean (SD)**	**Mean (SD)**	**Mean (SD)**
Age of child (years)	8.9 (4.7)	8.6 (4.6)	8.8 (4.6)
Age at seizure onset (years)	2.8 (3.7)	4.2 (4.1)	3.3 (3.9)
Number of current AED(s)	2 (1.3)	2 (0.9)	2 (1.2)
Number of past AED(s) tried	7.6 (6.8)	3.6 (5.6)	6.1 (6.6)

AEDs = antiepileptic drugs; C = Chromosome; DNM1 = Dynamin 1; KCNQ2 = Potassium voltage-gated channel subfamily Q member 2; PCDH19 = Protocadherin 19; SCN1A = Sodium voltage-gated channel alpha subunit 1; SCN2A = Sodium voltage-gated channel alpha subunit 2; SCN9A = Sodium voltage-gated channel alpha subunit 9; TSC2 = Tuberous Sclerosis Complex 2. *Due to rarity of some of the genetic mutations, descriptive statistics for positive mutations are not segregated by cannabis use history to maintain participant confidentiality.

### Reasons for using cannabis extracts

A significantly greater proportion of families with a history of cannabis extract use (35/41, 85%) had a child diagnosed with treatment-resistant epilepsy relative to non-using families (12/24, 50%) X^2^ (1, *N* = 65) = 9.5, *p* = 0.004 (Table 1). Similarly, using families reported a history of poorer antiepileptic drug efficacy (defined as <50% perceived reduction in seizures) (26/41, 63%) relative to non-using families (5/24, 21%), X^2^ (1, *N* = 65) = 9.5, *p* = 0.003. Using families also reported a significantly greater average number of past antiepileptic drugs tried and stopped (7.6 ± 6.8; range 0–30), compared to 3.6 ± 5.6 (range 0–21) for non-using families, *t*(61) = 0.31, *p* = 0.014. The average number of current antiepileptic drugs did not differ significantly between using families and non-using families, *t*(63) = 0.024, *p* = 0.98. There was no significant difference in the proportion of families reporting their child experiencing episodes of status epilepticus between using (31/41, 76%) and non-using families (14/24, 58%), X^2^ (1, *N* = 63) = 1.9, *p* = 0.25. Reasons for using, stopping, or for never using cannabis extracts are presented in Table 2.

**Table 2:** Descriptive statistics of themes for parent’s reason for using or not using cannabis extracts to treat their child’s epilepsy.

	All reasons N (%)	Main reason N (%)
**Currently using**	**n** = **34**	
*Reasons for starting cannabis extracts*		
*Uncontrolled seizures & concerns for child’s well-being	25 (74%)	20 (58%)
Intolerable antiepileptic drug side-effects	15 (44%)	4 (12%)
Success stories (media) and word of mouth	18 (53%)	4 (12%)
Personal research into cannabis extracts for epilepsy	9 (27%)	2 (6%)
To find natural alternative to pharmaceutical drugs	2 (6%)	2 (6%)
To manage other health conditions in addition to epilepsy	2 (6%)	1 (3%)
To try as a rescue medication	1 (3%)	1 (3%)
**Previously using and stopped**	**n = 7**	
*Reasons for stopping cannabis extracts*		
Cannabis extract was ineffective at reducing seizures	3 (43%)	2 (29%)
Parent noticed side-effects after starting cannabis extracts	4 (57%)	—
Problems with supply and/or access	2 (29%)	2 (29%)
Parent unsure how to use cannabis extracts	2 (29%)	1 (14%)
Concerned over quality of composition	1 (14%)	—
Child too young/too difficult to administer oil	1 (14%)	1 (14%)
Reported to child protection and the police	1 (14%)	1 (14%)
Parent noticed possible drug interactions	1 (14%)	—
**Never used cannabis extracts**	**n = 24**	
*Reasons for not using cannabis extracts*		
Fear of legal consequences and risk of child protection	13 (54%)	8 (33%)
Unsure how to access it	11 (46%)	4 (17%)
Concerned over safety risks and composition	8 (33%)	5 (21%)
I don’t know enough about it (dosage)	4 (17%)	4 (17%)
Need for medical supervision	2 (8%)	1 (4%)
Health provider advised against it	2 (8%)	—
Seizures currently well-controlled	2 (8%)	2 (8%)
Lacks evidence for use in a specific type of epilepsy	1 (4%)	—

*“Uncontrolled seizures & concerned for child’s well-being” captured the following themes: parent’s concerns over risk of sudden unexpected death in epilepsy (SUDEP), frequent hospitalizations due to seizures and status epilepticus episodes, and parent perceived deterioration in child’s physical health and/or cognition due to epilepsy.

Parental use of cannabis was similar across groups. The proportion of parents who self-reported using cannabis themselves at least once (for either medical or recreational reasons) did not significantly differ between families using cannabis extracts for their child (18/41, 44%) and those who did not (14/24, 58%), X^2^ (1, *N* = 64) = 1.07, *p* = 0.44. Of those families who had used cannabis at least once, similar proportions had used cannabis (for medical or recreational reasons) in the past 12 months across using (8/18, 44%) and non-using (4/14, 29%) families, X^2^ (1, *N* = 31) = 0.595, *p* = 0.48.

### Access and supply of cannabis extracts for use in epilepsy

**Figure 2:**
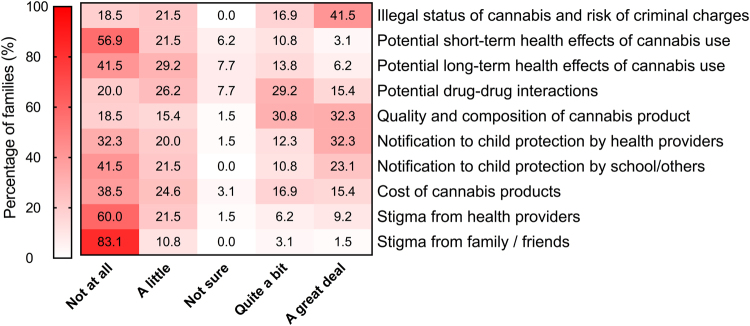
Heat map depicting family’s concerns regarding the use of cannabis extracts as a way to manage their child’s epilepsy. Each box indicates the percentage of all families who participated in the study (n = 65) rating their level of concern to a specific statement (abbreviated on the right-hand side) on a 5-point rating scale.

Of those who were using cannabis extracts, 27/41 families (66%) reported that it was relatively easy to obtain the cannabis extract. The same proportion of families also reported product supply as reliable, although 30/41 families (73%) reported worrying about ongoing supply. Of those who had tried cannabis extracts: six (15%) reported police involvement, arrests and/or charges due to possessing cannabis extracts; three (7%) families had cannabis extracts confiscated; four (10%) families were reported to child protection (cases later dropped); and six (15%) families reported difficulties with travel due to possession of cannabis extracts. Concerns regarding cannabis use in Australia are represented in Fig. 2 for all participating families.

Thirty-one (76%) families had disclosed to one or more of their doctors that they were using cannabis extracts to manage their child’s epilepsy. Of these, 14/31 families (45%) felt that they were receiving an adequate level of support from most of their health care providers, 11/31 families (35%) reported limited support, and 9/31 families (29%) reported inadequate support.

Most using families had tried 1–2 cannabis extracts (31/41, 76%) over a 12-month period at time of interview. A total of 25/41 (61%) of using families obtained their product free or “by donation”, while for the remaining families, the least and most amount of money spent on one month’s worth of cannabis product on average was $162 (range $10–$400) and $270 (range $15–$750), respectively. For those who paid for their cannabis extracts, 7/16 families (44%) who reported earning less than the median household income (<$75,000^57^) also reported that the cost of the cannabis product placed a significant strain on their finances compared to three (19%) families who reported earning over the median household income (*p* = 0.01, two-sided Fisher’s exact test).

### Preferred route of administration and method of access to medicinal cannabis

When asked the question, “If medicinal cannabis was legally available...”, oral administration of a liquid (oil) was the preferred route of administration for the majority of families (44/65, 68%), while the preferred method of access was via a pharmacy, as with any other medication (52/65, 80%). Preferences for route of administration and method of access did not statistically differ between using and non-using families (all *p* > 0.05). The remaining preferences for route of administration and mode of access are listed in Table 3.

**Table 3:** Preferred routes of administration and mode of access to cannabis-based extracts if medicinal cannabis became legally available (N = 65).

Preferred routes of administration	N (%)
Oral use - Liquid (e.g. oil)	44 (67.7%)
Oral use - Tablets/capsules (swallowed)	21 (32.3%)
I do not care as long as it is effective	18 (27.7%)
Oral use - Chewable tablet	11 (16.9%)
Mouth spray	9 (13.8%)
Via the nose - intranasal spray	6 (9.2%)
Via the nose - Inhalant (e.g. like “Vick’s vapor”)	5 (7.7%)
Skin patches	5 (7.7%)
Edible (e.g., butter/resin, tea infusion)	2 (3.1%)
Smoked – Joint	2 (3.1%)
Vaporiser	2 (3.1%)
Suppository	2 (3.1%)
*Other*: Raw cannabis material for juicing	1 (1.5%)
*Other*: Slow-release tablet administered once daily	1 (1.5%)
Smoked - Water pipe (“bong”)	—
Preferred mode of access	
From a pharmacy like other medication	52 (80%)
From a special licensed cannabis dispensary	16 (24.6%)
I do not care as long as there is regular supply	9 (13.8%)
Grow or make your own	7 (10.8%)
From a supplier	5 (7.7%)
Trade/buy from a friend	2 (3.1%)
No opinion	1 (1.5%)
*Other*: Sold as a supplement	1 (1.5%)

Respondents were asked to choose their preferred routes of administration and access models from a list containing several possibilities therefore sum of responses do not add to 100%.

### Sources and types of cannabis extracts

Sixty-one cannabis extract samples were collected from 38/41 families who were currently using or had previously used cannabis for their child’s epilepsy (refer to flow diagram in Fig. 1). Of the individual extracts, 38/61 (62%) had been sourced from a local “medicinal cannabis” supplier, 9/61 (15%) were obtained from a dealer of recreational cannabis, 8/61 (13%) were made at home by the family, 4/61 (7%) from an international online supplier, 2/61 (3%) were accessed via federal/state government schemes, and 1/61 (2%) from an Australian online supplier. In terms of composition, 51/61 (84%) were oil-based, 4/61 (7%) were alcohol-based (6%), 3/61 (5%) were thick pastes, 1/61 (2%) was a nasal spray, 1/61 (2%) was an oral spray and 1/61 (2%) was compressed trichomes (‘hash’). The majority of families did not know how their product was made (37/61, 61%), while the remaining reported the following methods: 18/61 (30%) were ‘oil-based low heat extraction’, 3/61 (5%) ‘alcohol-based cold extraction’, 2/61 (3%) ‘alcohol-based low heat extraction’, and 1/61 (2%) ‘dry ice sift method’.

If the family were co-administering two or more cannabis extracts to their child simultaneously at the time of interview, a combined dose was calculated by merging data for each individual cannabis extract. This resulted in a final dataset of 51 cannabis extracts. All merged samples will hereafter be simply referred to as cannabis extracts. The median length of time using all cannabis extracts was 3 ± 23.5 months (range 1 week–13 years). The majority of cannabis extracts were administered orally (42/51, 82%) either into the mouth (38/51, 75%) or under the tongue (4/51, 8%). Some were also administered with food (12/51, 24%). None were smoked or vaporised. Six cannabis extracts were administered via percutaneous endoscopic gastrostomy (PEG) feed (6/51, 12%) and one was applied topically. Most products did not have an expiry date that the family was aware of. For the majority of cannabis extracts (36/51, 71%), families reported expecting CBD to be the main active ingredient. Of these, 13/36 (36%) cannabis extracts were also expected to contain THC.

### Perceived efficacy, side effects and duration of use

Families rated their child’s condition on the PGIC a median of ‘2’ indicating “much improved”. When the family was asked about the percentage change in average seizure frequency since starting the cannabis extract, 26/51 (51%) cannabis extracts were associated with an average seizure reduction of 75–100%, 5/51 (10%) with a 50–75% reduction, none with a 25–50% reduction, 2/51 (4%) with a 0–25% reduction, 10/51 (20%) resulted in “no change”, and 4/51 (8%) were associated with an increase in seizures (see Table 4). Only one family reported complete seizure-freedom in their child for at least 12 months after starting cannabis extracts. Using criteria outlined in the methods, a total of 38/51 (75%) cannabis extracts were grouped as perceived “effective” while 13/51 (25%) were grouped as perceived “ineffective”. Twenty-two (43%) cannabis extracts were associated with families reducing some, but not all, of their child’s concomitant antiepileptic medication while 26/51 (51%) cannabis extracts did not result in any changes to the child’s current medication (Table 4). Three (6%) cannabis extracts were associated with complete cessation of all antiepileptic drugs. Using the Wilcoxon Signed-ranks Test, we identified no significant difference in the median number of antiepileptic drugs the child was on before (2, range 0–6) and after (1.5, range 0–4) starting cannabis extracts that were perceived “effective” (*Z* = −1.9, *p* = 0.06) nor in the median number of antiepileptic drugs the child was on before (1, range 0–6) and after (3, range 0–6) starting cannabis extracts that were perceived “ineffective” (*Z* = −1.7, *p* = 0.08).

**Table 4:** Descriptive statistics of parent-reported perceived efficacy of cannabis extracts.

	“Effective” cannabis extracts	“Ineffective” cannabis extracts	Total
	N (%)	N (%)	N (%)
	38	13	51
Adapted Patient Global Impression of Change (PGIC) scale			
1 Very much improved	23 (61%)	—	23 (45%)
2 Much improved	10 (26%)	—	10 (20%)
3 Minimally improved	5 (13%)	—	5 (10%)
4 No change	—	7 (54%)	7 (13%)
5 Minimally worse	—	2 (15%)	2 (4%)
6 Much worse	—	3 (23%)	3 (6%)
7 Very much worse	—	1 (8%)	1 (2%)
Perceived change in seizure frequency			
75–100% reduction	26 (68%)	—	26 (51%)
50–75% reduction	5 (13%)	—	5 (10%)
25–50% reduction	—	—	—
0–25% reduction	2 (5%)	—	2 (4%)
No change	1 (3%)	9 (69%)	10 (20%)
Increase in seizures	—	4 (31%)	4 (7%)
*No rating*: Emergency medication only	2 (5%)	—	2 (4%)
*No rating*: Child experiencing infrequent seizures	1 (3%)	—	1 (2%)
*No rating*: EEG activity improved but not seizures	1 (3%)	—	1 (2%)
Reduction in child’s antiepileptic drug(s) after starting cannabis extracts			
Yes, all	3 (8%)	—	3 (6%)
Some medication but not all	22 (58%)	—	22 (43%)
No, none	13 (34%)	13 (100%)	26 (51%)

Parent-rated perceived efficacy of 51 cannabis extracts on the adapted Patient Global Impression of Change (PGIC) scale of change in child’s overall condition, a rating of valid percentage change in seizure frequency, and changes to the child’s antiepileptic drug regimen after commencing cannabis extracts.

 All cannabis extracts were first initiated as adjunctive treatments, except in one case where the family reported use as a first-line treatment. Families reported using a median of two (range 0–6) antiepileptic drugs for their child’s epilepsy in addition to the cannabis extract, with the most frequently used antiepileptic drugs being sodium valproate (14/51, 28%), clobazam (14/51, 28%) and levetiracetam (9/51, 18%).

Thirty-three (65%) cannabis extracts were reported to have other beneficial health effects on their child. These included improvements in cognition (18/51, 35%), emotional well-being (16/51, 31%), language skills (12/51, 24%), social activity (7/51, 14%), physical activity (11/51, 22%), sleep (9/51, 18%), pre-existing gastrointestinal symptoms (5/51, 10%), behaviour (2/51, 4%), dystonia (1/51, 2%), and eczema (1/51, 2%).

While no serious adverse effects were reported, 19/51 (37%) cannabis extracts were reported to have side effects. These included worsening of pre-existing problem behaviours (6/51, 12%), possible increase in seizures (6/51, 12%), drowsiness/lethargy (4/51, 8%), changes to appetite or weight (3/51, 6%), gastrointestinal upset (3/51, 6%), possible intoxication (2/51, 4%), and temporary changes in sleep and mood (2/51, 4%). Of these, three cannabis extracts were later discontinued.

No significant differences in behaviour as measured via the CBCL were identified for children who were using cannabis extracts versus those who were not using (total problems score: ages 1.5–5 years: *t*(19) = 0.35, *p* = 0.732; ages 6–18 years: *t*(33) = 1.31, *p* = 0.20). The number of children at risk for behaviour problems (defined as *T* score ≥ 65) did not significantly differ between those who were using cannabis extracts versus those who were not (all *p* > 0.05). There were also no significant differences in parent-reported child between families who were using cannabis extracts versus those who were not (TAPQOL all subscales, *p* > 0.2; QOLCE-55 all subscales: *p* > 0.3) (see Supplementary Tables 2, 3).

### Cannabinoid profiles of extracts

**Figure 3:**
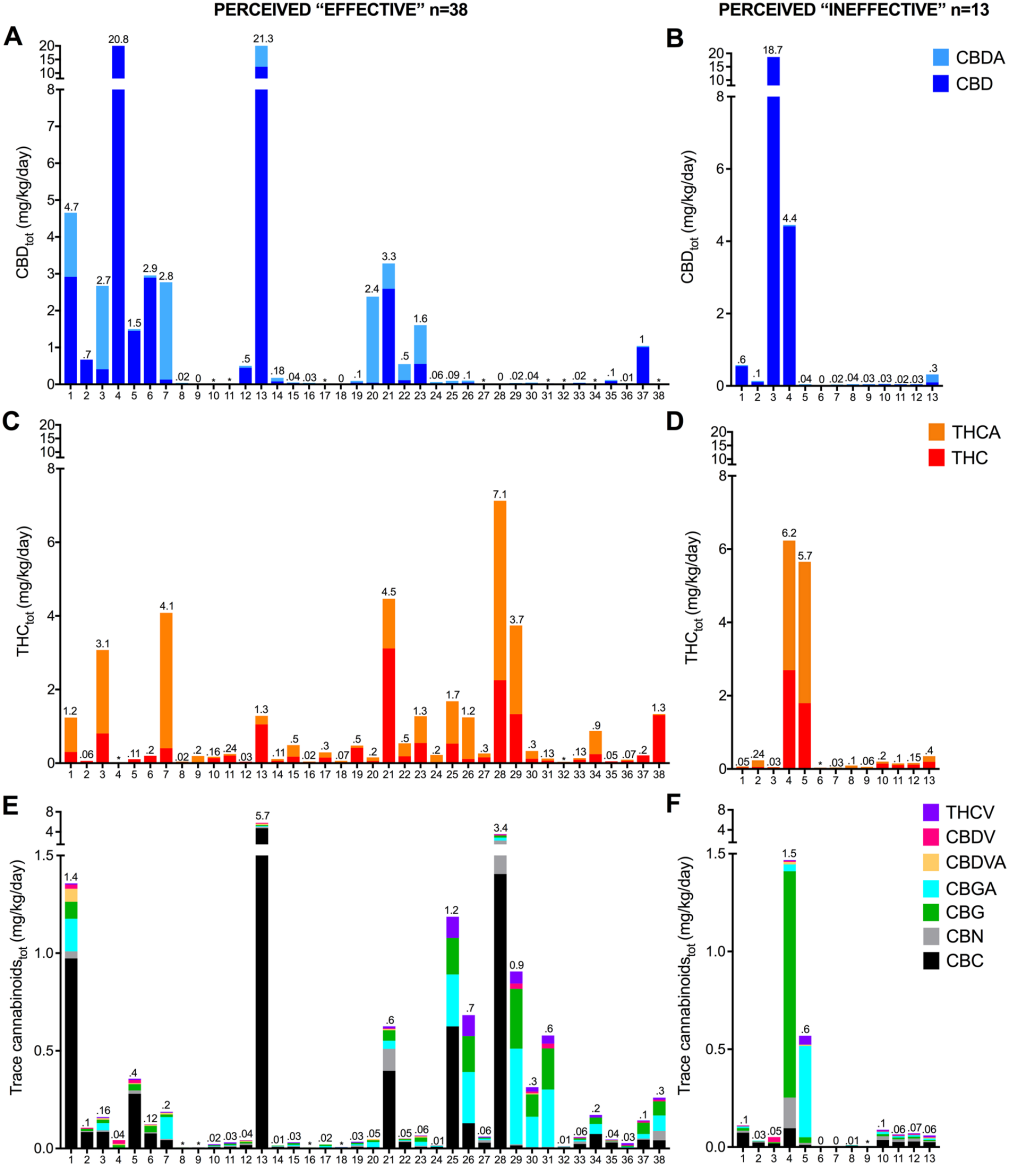
Dose of cannabinoids found in individual cannabis extract samples that were perceived “effective” or “ineffective” according to the family. Dose of cannabinoids in 51 cannabis extract samples that were being used by families to treat their child’s epilepsy. Samples perceived “effective” (**A**,**C**,**E**) and “ineffective” (**B**,**D**,**F**) are depicted in separate graphs. Dose of CBD and CBDA **(A**,**B)**, THC and THCA (**C**,**D**), and trace cannabinoids (**E**,**F**) are depicted in milligrams (mg) per kilogram (kg) of child’s body weight per day. Note the difference scale for the Y-axis between graphs. Values above each individual column indicate total cannabinoid content for that graph (mg/kg/day).

The individual cannabinoid profiles of “effective” and “ineffective” extracts are presented in Fig. 3 as daily cannabinoid dose relative to body weight (i.e. mg/kg/day). The body weight of the children ranged from 8–90 kg. Cannabinoid profiles varied enormously, clearly reflecting the unstandardized nature and chemovar-to-chemovar variability of artisanal preparations (Fig. 3). Overall, there were no significant differences in the cannabinoid content of extracts perceived as “effective” compared to those perceived as “ineffective” (*p* > 0.1 for all individual cannabinoids), nor in total mg/kg/day cannabinoid content (*p* = 0.9) (Table 5).

**Table 5:** Descriptive statistics of the type and dose of cannabinoids and terpenoids in cannabis extracts based on perceived efficacy.

**Cannabinoids**	Cannabis extracts perceived “effective” (N = 38)				Cannabis extracts perceived “ineffective” (N = 13)	
	N (Present)	Mean (SD) (mg/kg/day)	Range	N (Present)	Mean (SD) (mg/kg/day)	Range
CBD	37	1.23 (3.87)*	0–20.81	10	1.84 (5.20)*	0–18.7
THCA	36	0.56 (1.08)	0–4.97	13	0.62 (1.37)	0.004–3.9
CBDA	36	0.55 (1.58)	0–9.0	12	0.03 (0.06)	0–0.22
THC	38	0.39 (0.65)	0–3.12	12	0.40 (0.84)	0–2.7
CBC	38	0.24 (0.80)	0–4.74	10	0.03 (0.03)	0–0.1
CBN	32	0.04 (0.14)	0–0.86	8	0.02 (0.04)	0–1.16
CBGA	36	0.07 (0.14)	0–0.60	10	0.04 (0.13)	0–0.47
CBG	37	0.06 (0.10)	0–0.42	10	0.10 (0.32)	0–1.16
CBDVA	24	0.01 (0.05)	0–0.29	7	0.002 (0.004)	0–0.013
CBDV	19	0.01 (0.02)	0–0.08	7	0.003 (0.008)	0–0.03
THCV	35	0.01 (0.03)	0–0.11	8	0.007 (0.013)	0–0.05
**Terpenoids**	**N (Present)**	**Mean (SD) (μg/kg/day)**	**Range**	**N (Present)**	**Mean (SD) (μg/kg/day)**	**Range**
β-caryophyllene	36	50.6 (104)	0–497.9	11	23.5 (53.8)	0–182.4
caryophyllene oxide	29	13.5 (53.97)	0–329.5	10	4.5 (13.5)	0–49.2
β -myrcene	31	16.6 (33.6)	0–123.6	10	7.8 (21.8)	0–80
d-limonene	30	5.9 (10.3)	0–46.5	7	3.1 (8.2)	0–30
humulene	33	19.7 (37.4)	0–140.4	8	4.8 (11.5)	0–39.8
α-pinene	29	10.7 (21.5)	0–98.2	6	7.1 (20.1)	0–73
β-linalool	21	3.14 (7.45)	0–34.9	6	1.2 (2.6)	0–8
α-bisabolol	26	9.93 (30.4)	0–178	7	1.46 (4.09)	0–15
guaiol	23	4.97 (18.4)	0–112.2	6	0.4 (0.75)	0–2.11
β-pinene	27	3.26 (5.28)	0–18.02	8	2.23 (6.86)	0–25
nerolidol	25	3.3 (7.3)	0–31.9	7	1.68 (4.04)	0–14.32
terpinolene	17	2.34 (7.02)	0–37.8	6	0.05 (0.13)	0–0.48
camphene	6	0.11 (0.34)	0–1.56	5	0.19 (0.55)	0–2
ocimene	21	4.04 (12.06)	0–52.62	5	0.26 (0.69)	0–2.44
y-terpinene	8	0.62 (1.76)	0–8.68	4	0.02 (0.06)	0–0.21
p-cymene	8	0.34 (1.04)	0–4.59	4	0.59 (1.88)	0–6.79
3-carene	27	1.67 (5.29)	0–32.28	8	0.95 (1.68)	0–5.02
1-8-cineol	11	1.02 (3.96)	0–19.37	5	0.3 (0.71)	0–2.49
α-terpinene	8	0.33 (1.36)	0–8.28	5	0.03 (0.06)	0–0.05
geraniol	5	0.19 (0.59)	0–2.81	3	0.003 (0.005)	0–0.012
isopulegol	2	0.08 (0.28)	0–1.42	0	—	—

Average dose of individual cannabinoids and terpenoids in 38 cannabis extracts perceived “effective” and 13 cannabis extracts perceived “ineffective” by the family in treating their child’s seizures.

*Both datasets (perceived “effective” and “ineffective” group) each contained one 98% pure, pharmaceutical-grade CBD product that was being accessed legally via a government scheme. Excluding these two data points resulted in an average CBD dose of 0.69 ± 2.12 (range 0–12.3) mg/kg/day for perceived “effective” and 0.44 ± 1.26 (range 0–4.4) mg/kg/day for perceived “ineffective”. Presence in the cannabis extract sample was defined as >1 ng/mg for both cannabinoids and terpenoids.

Most families reported expecting CBD-dominant cannabis extracts; in reality, however, only 3/51 extracts (6%) provided doses of CBD that approached the minimum doses used in recent clinical trials of CBD (i.e. ≥ 10 mg/kg/day)^58^. Indeed, the average dosage of CBD for all samples was 1.38 ± 4.2 (range 0–20.8) mg/kg/day. While CBD appeared to the most highly dosed cannabinoid across all of the samples (see Table 5), this dataset included two high-dose, pharmaceutical-grade (98% pure) CBD products that were being legally accessed via a government scheme. When excluding these two data points, the average CBD dose fell to 0.64 ± 1.94 (range 0–12.3) mg/kg/day. THCA and THC ranked as the first and second most prevalent cannabinoids (17/51, 33% and 14/51, 27.5%, respectively), while CBD was most prevalent cannabinoid in just under a quarter of samples (12/51, 23%) and was undetectable in 4/51 samples (8%).

Indeed, 98% of all cannabis extract samples contained THC and/or Δ^9^-tetrahydrocannabinolic acid (THCA) with an average THC_tot_ (combination of THC and THCA) of 0.97 ± 1.7 (range 0–7.1) mg/kg/day. The mean dosage of THC for all samples was 0.39 ± 0.7 (range 0–3.1) mg/kg/day, with 41 (80%) samples containing less than 0.5 mg/kg/day of THC. Levels of other trace phytocannabinoids (listed in Fig. 3) tended to be low and only detectable in a portion of samples, with an average total dosage of trace cannabinoids of 0.38 ± 0.95  (range 0–5.68) mg/kg/day.

### Terpenoid profiles of extracts

The average total dosage of terpenoids was 128.8 ± 222.8 (range 0–1,087) μg/kg/day. The three most highest dosed terpenoids were β-caryophyllene (28/51 samples, 54.9%), β-myrcene (12/51 samples, 23.5%), and α-pinene (4/51 samples, 7.8%) (see Supplementary Fig. S1 for further detail). The average dose of these were 43.68 ± 94.04 (range, 0–497.94) μg/kg/day and 14.31 ± 31.05 (range 0–123.64) μg/kg/day, respectively. No significant differences were identified in extracts perceived “effective” and “ineffective” (*p* > 0.1 for all individual terpenoids), nor in total mg/kg/day terpenoid content (*p* = 0.3). Full terpenoid dosage data are available in Table 5.

### Urinalysis

Of the 65 participants interviewed, urine samples were collected from 26/41 children from currently using families and 21/24 children from non-using families. There were no cannabinoids or metabolites detected in any of the urine samples acquired from families who had previously used or had never used (all cannabinoid concentrations less than limit of detection). Mean urinary concentrations of cannabinoids and/or metabolites (creatinine-adjusted) varied between children as would be expected from the diversity of extracts and doses being used: THC-COOH, 258 ± 575 ng/mL; 11-OH-THC, 63 ± 128 ng/mL; CBD, 79 ± 224 ng/mL; CBG, 53 ± 152 ng/mL; THCA, 3.5 ± 11 ng/mL; THC and CBN not detected. Reported use of cannabis extracts by currently using families was confirmed in all but two urine samples: these two samples belonged to children who were receiving cannabis extracts of relatively low cannabinoid content (0.053 and 0.184 mg/kg/day of total cannabinoids, respectively). While the average THC metabolite concentrations were relatively low, in some children these concentrations were quite high. For example, two children exhibited THC-COOH urinary concentrations as high as 1,947 ng/mL and 2,228 ng/mL, respectively. No significant differences in urinary concentrations of cannabinoids or their metabolites were identified between samples perceived “effective” and “ineffective” (*p* < 0.05).

## Discussion

The current study had two major aims. The primary aim was to examine the motivations and experiences of families using cannabis extracts to treat a child suffering from epilepsy relative to families who were relying only on conventional treatments. A secondary aim was to provide a detailed analysis of the composition of cannabis extracts that were perceived as ‘effective’ versus those perceived as ‘ineffective’ in reducing seizure frequency. While the study was not designed to determine efficacy of these extracts in a conventional sense, it was hoped that some clues as to effective cannabinoid treatments for epilepsy might emerge from such an analysis.

In agreement with our earlier survey^13^, families using cannabis extracts to treat their child’s epilepsy were more likely to report their child as being “treatment-resistant”, or in some cases, having experienced intolerable side-effects. The main concerns for families using cannabis extracts for their child’s epilepsy was its current illegal status and the unknown quality and composition of illicit preparations. These concerns were also reported by non-using families. Less worrisome considerations included stigma from health professionals or family and the potential short-term and long-term effects of cannabis use on the child. The latter may reflect the family’s desperate attempt to find an effective treatment for their child’s seizures, a decision that may override any possible unknown health risks of the cannabis extract on the child. Most families reported that it was relatively easy to obtain illegal cannabis extracts, yet still worried about ongoing supply. Most families reported wanting to access cannabis-based products from a pharmacy similar to other medication suggesting a preference to legitimise cannabis-based products as opposed to seeking an ‘alternative’ treatment. The majority of families had disclosed their use of cannabis extracts to their treating doctor with substantial variation in the level of support provided to families by the medical profession.

A considerable proportion of families reported cannabis extracts being “effective” in reducing their child’s seizure burden and improving their overall condition, with one family reporting seizure-freedom in their child for at least 12 months. Over half of the cannabis extracts were associated with families reducing or ceasing their use of the child’s conventional antiepileptic drugs. Contrary to the expectation of families, the majority of cannabis extracts perceived “effective” contained low or negligible concentrations of CBD, comparable with the low concentrations of CBD found in Australian street cannabis, which is almost uniformly THC-dominant^35^. The minimum dose described in clinical trials of purified CBD for severe childhood epilepsies (10 mg/kg/day)^58^ was reached with only 6% of samples in the present study. This raises questions concerning previously published articles reporting efficacy of what families believed to be ‘CBD-dominant’ cannabis products^10,11^ and emphasises the need for chemical analysis of extracts in such studies^59^.

Correspondingly, several preclinical studies^38–40^ have demonstrated dose-dependent anticonvulsant effects of THC although others have also shown that THC could potentially provoke seizures directly at higher doses, or during withdrawal from THC^41–43^. On the other hand, ultra-low dose THC (range 0.04–0.12 mg/kg/day, dronabinol) had anticonvulsant effects in a case series of six children with epilepsy^29^. More recently, a multi-site, retrospective study of 74 children with treatment-resistant epilepsy demonstrated promising effects of a standardised preparation of CBD and THC (ratio of 20:1) on seizure frequency for at least 3 months^25^. Here the THC dose was carefully controlled so as not to exceed 0.5 mg/kg/day. These findings are now being extended in a Phase I open-label clinical trial^60^. Taken together, these observations suggest possible utility of low doses of THC as an adjunct to current antiepileptic drug treatment and/or in combination with other cannabinoids such as CBD. In the present study, the mean dose of THC was 0.39 ± 0.7 mg/kg/day, with 80% of children using cannabis extracts containing less than 0.5 mg/kg/day of THC. However, despite THC being present in nearly all of the extracts analysed, concentrations did not differ between samples perceived as “effective” and “ineffective”. Nonetheless, concerns remain over the effects of THC on the developing brain, with some children exhibiting THC metabolite urinary concentrations, levels that would be associated with high plasma THC levels, intoxication and possible impairment in adults^61^. Further research is needed to better understand the dose-dependent effects of THC on seizure susceptibility and the developing brain.

Preclinical reports indicate anticonvulsant effects of a number of other phytocannabinoids, including CBDA^19^, CBDV^18^ and Δ^9^–tetrahydrocannabivarin (THCV)^17^, as well as terpenoids such as β-caryophyllene in high doses^37^. The present study was unable to link these specific trace cannabinoids or terpenoids to “effective” samples, reflecting the pool of THC- and THCA-dominant extracts that was uncovered. β-caryophyllene has recently been shown to protect against PTZ- induced seizures in a preclinical model of epilepsy, but at much higher doses than were found in the extracts analysed here^37^.

The design of the current study precludes any definitive statements on the efficacy of cannabis extracts and its components. Limitations include reliance on the retrospective nature of family self-report, possible participant bias, lack of clinician-confirmed epilepsy diagnosis, and the inherent subjectivity of seizure counting, particularly in the absence of a seizure diary^62^. Regression to the mean in seizure reporting, as well as seizure fluctuation are also cited as possible confounds in epilepsy research^63,64^. Psychosocial outcomes and seizure activity can fluctuate over a relatively short period of time as a child matures and thus may be incorrectly perceived as a result of cannabis extract treatment. Family-reported observations of global change in their child’s condition following cannabis use may be unreliable in the absence of formal pre- and post-outcome measures. This may particularly be the case for psychosocial measures such as child behaviour and quality of life for which we did not find clear differences in in the present study.

Placebo effects appear to be strong in epilepsy trials^65^ and may be particularly pertinent to medicinal cannabis use, which has attracted intense media coverage of specific cases and community advocacy, leading to high expectations of success in some parents. Indeed, a two-fold greater placebo response in children compared to adults has been reported^66^. Relevant to our sample, patients with intellectual disabilities and severe epilepsy may be particularly liable to a placebo response^67^. Carer ratings of children’s behavioural or cognitive response to cannabis treatment may be prone to “placebo by proxy”, whereby the child may react to changes in how the caregiver is interacting with and monitoring them^68,69^. Difficulties accessing a cannabis product may also impact ratings of cannabis efficacy^12,70^ and it is conceivable that participation was positively skewed towards those who benefitted from the cannabis product versus those who had not. Finally, the present study was not sufficiently powered to identify statistical differences between cannabis extract samples perceived “effective” and “ineffective”, particularly given the low number of the latter type of sample.

Despite these limitations, the present study throws a spotlight on the world of families who are resorting to the use of illicit cannabis extracts to treat their child’s epilepsy. This underlines the huge unmet clinical need in the management of treatment-resistant epilepsy in childhood. These findings warrant further investigation into the added value of specific cannabinoids, alone and in combination with other cannabinoids and standard antiepileptic drugs, in treatment-resistant epilepsy.

## Electronic supplementary material

Supplementary Material
