# Author Correction: Composition and Use of Cannabis Extracts for Childhood Epilepsy in the Australian Community

**DOI:** 10.1038/s41598-018-30164-8

**Published:** 2018-08-02

**Authors:** A. Suraev, N. Lintzeris, J. Stuart, R. C. Kevin, R. Blackburn, E. Richards, J. C. Arnold, C. Ireland, L. Todd, D. J. Allsop, I. S. McGregor

**Affiliations:** 1 The Lambert Initiative for Cannabinoid Therapeutics, School of Psychology, The University of Sydney, Sydney, 2050 Australia; 2 Addiction Medicine, Central Clinical School, Faculty of Medicine, The University of Sydney, Sydney, 2006 Australia; 3 The Langton Centre, Drug and Alcohol Services, South East Sydney Local Health District, NSW Health, Surry Hills, 2010 Australia; 4 Department of Pharmacology, Faculty of Medicine, University of Sydney, Sydney, NSW 2006 Australia; 5 Epilepsy Action Australia, Sydney, Australia

Correction to: *Scientific Reports* 10.1038/s41598-018-28127-0, published online 05 July 2018

This Article contains an error in the Methods section under subheading ‘Participants and semi-structured interview’,

“The interview was delivered by a female psychologist (A.S. or R.B.) to one or both parents/guardians. Interviews were conducted either at the participant’s home, or at a private clinic room at the Brain and Mind Centre, Sydney or the Centre for Children’s Health Research, Brisbane, and lasted approximately 1.5 hours.”

should read:

“The interview was delivered by a female psychologist (A.S., R.B. or E.R) to one or both parents/guardians. Interviews were conducted either at the participant’s home, or at a private clinic room at the Brain and Mind Centre, Sydney or the Centre for Children’s Health Research, Brisbane, and lasted approximately 1.5 hours.”

