# Supplementary material for: Composition and Use of Cannabis Extracts for Childhood Epilepsy in the Australian Community
Source: Sci Rep. 2018 Jul 5;8:10154. doi: 10.1038/s41598-018-28127-0 (PMC6033872; doi:10.1038/s41598-018-28127-0)
Supplement: Supplementary file 1 — Supplementary Material [file 41598_2018_28127_MOESM1_ESM.docx]

**Supplementary Information**

**Composition and Use of Cannabis Extracts for Childhood Epilepsy in the Australian Community**

Suraev A.S.^1^, Lintzeris N.^2,3^, Stuart J.^1^, Kevin R.C.^1^, Blackburn R.^1^, Richards E.^1^, Arnold, J.C.^1,4^, Ireland C.^5^, Todd L.^5^, Allsop D.J.^1^, & McGregor I.S.^1,*^

^1^The Lambert Initiative for Cannabinoid Therapeutics, The University of Sydney, School of Psychology, Sydney, 2050, Australia

^2^Addiction Medicine, Central Clinical School, Faculty of Medicine, The University of Sydney, Sydney, 2006, Australia

^3^The Langton Centre, Drug and Alcohol Services, South East Sydney Local Health District, NSW Health, Surry Hills, 2010, Australia

^4^Department of Pharmacology, Faculty of Medicine, University of Sydney, NSW, 2006, Australia

^5^Epilepsy Action Australia, Sydney, Australia

**Supplementary Methods S1**

**Survey**

**DEMOGRAPHICS**

1. **Relationship of person filling out this survey to the child (with epilepsy):** [Multiple choice] Parent/Guardian; Sibling; Grandparent; Other
2. If selected 'Other', please specify:
3. **Child's gender:** [Multiple choice] Male; Female; Other
4. **Child's date of birth:** DD/MM/YYYY
5. **Body weight of child:** [Free-text] _____ kg
6. **Height of child (cm):** [Free-text] _____ cm
7. **Number of children in the family:** [Free-text] ______
8. **If more than one child, where does your child with epilepsy fit in the birth order?** (E.g. If your child is 3rd of 4 children, please write '3') [Free-text] _____
9. **Who are the main carers (please state relationship or role) of your child with epilepsy over the course of an average week?** [Multiple choice]: Mother; Father; Grandparent; Carer; Other
10. **What is your household's income per year?** [Multiple choice] Less than $25,000; $25,000 to $50,000; $50,000 to $75,000; $75,000 to $100,000; $100,000 to $125,000; $125,000 +

**CHILD MEDICAL HISTORY**

1. **At what age did your child's seizures begin?** [Free-text] _______ (Months)
2. **What is the epilepsy diagnosis or epilepsy syndrome of your child?**
3. **At what age did your child get diagnosed with epilepsy?** [Free-text] _______ (Months)
4. **Which health provider made the diagnosis?** [Multiple choice] Neurologist (general OR specialising in children or epilepsy); Paediatrician; GP; Other
5. **What is the cause of the epilepsy?** [Multiple choice] Genetic; Structural; Metabolic; Unknown.
6. **Has your child ever had genetic testing for their epilepsy?** [Multiple choice] Not tested; Tested but I do not know result or results were inconclusive; Tested but abnormal result of 'array CGH'; Tested and positive for a mutation on a gene
7. **Please specify the gene that tested positive:** [Free-text] _______
8. **Have you ever been told by your treating doctor that your child's epilepsy was intractable epilepsy, uncontrolled epilepsy, refractory epilepsy, medication-resistant epilepsy, or drug-resistant epilepsy?** Yes; No
9. **At what age was your child diagnosed with drug-resistant epilepsy**? [Free-text] _______ (Months)
10. **How would you describe your child's seizure frequency?** [Open-ended]
11. **What is the average number of seizures your child experiences over an average MONTH by type?** Tonic-clonic ("grand mal seizures") [free-text]; Clonic [free-text]; Tonic [free-text]; Absence (typical or atypical) [free-text]; Atonic; Myoclonic jerks (-atonic or -tonic) [free-text]; Focal ("simple partial") [free-text]; Focal dyscognitive ("complex partial") [free-text]; Infantile/epileptic spasms [free-text]; Unknown type [free-text]
12. **[Using families only] What is the average number of seizures your child experiences over an average MONTH by type since starting cannabis extracts?** Tonic-clonic ("grand mal seizures") [free-text]; Clonic [free-text]; Tonic [free-text]; Absence (typical or atypical) [free-text]; Atonic; Myoclonic jerks (-atonic or -tonic) [free-text]; Focal ("simple partial") [free-text]; Focal dyscognitive ("complex partial") [free-text]; Infantile/epileptic spasms [free-text]; Unknown type [free-text]
13. **Has your child ever experienced episodes of status epilepticus?** Yes; No
14. **If Yes, how long did these episodes last for?** [Multiple choice] Between 15 to 30 minutes in duration 30 minutes or longer Repetitive brief seizures occurring for more than 30 minutes duration without a recovery in between
15. **What was the longest duration of seizure-freedom in the 12 months prior to commencing current treatment?** [free-text] ______ (Months)
16. **How many prescribed antiepileptic drug(s) is your child with epilepsy on currently?** [Free-text] ______ (Numerical)
17. **How many prescribed antiepileptic drug(s) has your child with epilepsy tried and stopped?** [Free-text] ______ (Numerical)
18. **If you think back to the most effective conventional antiepileptic drug(s) (alone or in combination) that your child has tried, how would you rate its effectiveness in terms of % change in average seizure frequency?** [Free-text] ________ (Please write down a percentage figure from 0 - 100%)
19. **What treatments (other than anti-epileptic drugs) have you tried for your child, and do you consider these treatments to be successful in managing their epilepsy?** Vagus nerve stimulation [Multiple choice] Unsuccessful; No change; Successful; Never tried. Ketogenic diet [Multiple choice] Unsuccessful; No change; Successful; Never tried. Surgery [Multiple choice] Unsuccessful; No change; Successful; Never tried. Other [Multiple choice] Unsuccessful; No change; Successful; Never tried.
20. **Does anyone else in the family have a diagnosis of epilepsy?** Yes; No
21. **Relationship of family member with epilepsy to the child (with epilepsy):** (Please tick any that apply) Mother; Father; Sibling(s); Grandparent(s); Extended family member

**CHILD WELL-BEING AND SCHOOLING**

1. **Does your child attend school?** [Multiple choice] Mainstream school; Special education unit at mainstream school; Special education school; Home schooling; Does not attend school yet (too young); Does not attend school (unable to attend)
2. **If your child is unable to attend school, can you please explain why?** [Open-ended] ________
3. **What is your child's current year level?** [Multiple choice] Preschool; Kindergarten; Year 1; Year 2; Year 3; Year 4; Year 5; Year 6; Year 7; Year 8; Year 9; Year 10; Year 11; Year 12
4. **Did he or she ever repeat any grades?** Yes; No
5. **Which year(s) did he or she repeat?** [Choose any that apply] Preschool; Kindergarten; Year 1; Year 2; Year 3; Year 4; Year 5; Year 6; Year 7; Year 8; Year 9; Year 10; Year 11; Year 12
6. **In terms of level of achievement, how would you say your child is performing relative to other students in their grade?** [Multiple choice] Below average; Average; Above average
7. **How would you rate their school attendance during an average week?** [Multiple choice] < 50% (Attends less than half of the days per week); 50-75% (Attends just over half of the days per week); 75-95% (Attends most days with 1-2 days missed); > 95% (Attends almost every day with the occasional "sick day")
8. **Were there times when your child (with epilepsy) missed out on a significant amount of schooling?** Yes; No
9. **If yes, please provide the length of time and reason:** [Open-ended] ________
10. **If you are currently employed, to what extent does your child's epilepsy affect your work attendance?** [Multiple choice] Not at all; A little; Somewhat; Quite a bit; A great deal

**CANNABIS PRODUCT USE**

1. **Are you currently using cannabis products for treatment of your child's epilepsy?** Yes; No
2. **Why did you decide to start using cannabis products for your child's epilepsy**? [Open-ended] _____
   1. **Please choose the most important factor:** [Open-ended] ______
3. **Have you previously trialed cannabis products for your child's epilepsy and stopped?** Yes; No
4. **Why did you stop?** [Open-ended] ______
   1. **Please choose the most important factor:** [Open-ended] ______
5. **Why have you not tried cannabis products for your child's epilepsy to date?** [Open-ended] ______
   1. **Please choose the most important factor:** [Open-ended] ______
6. **At what age did your child with epilepsy start using cannabis products?** [Free-text] _______ (Months)
7. **How long has your child used cannabis products for?** [Free-text] _______ (Months)
8. **How easy or difficult do you find it to obtain the cannabis product?** [Multiple choice] Very easy; Somewhat easy; Not particularly easy or hard; Somewhat hard; Very hard
9. **How reliable was the supply of the cannabis product?** [Multiple choice] Very unreliable; Somewhat reliable; Not particularly reliable or unreliable; Somewhat reliable; Very reliable
10. **Did you ever worry about your supply of cannabis?** [Multiple choice] Not at all; Mostly no; Yes and no; Somewhat, yes; Yes, very much so
11. **If it did worry you, please expand on your response:** [Open-ended] ______
12. **Have you ever been in the situation of not being able to source the product?** Yes; No
13. **If yes, what happened?** [Open-ended] ______
14. **How many cannabis products has your child tried in total?** [Free-text] _______ (Number)
15. **How many different cannabis products have you tried for your child's epilepsy in the past year?** [Free-text] _______ (Number)
16. **How many different cannabis products have you tried for your child's epilepsy in the past 5 years?** [Free-text] _______ (Number)
17. **Was there ever a break of more than 1 month from using the cannabis product**? Yes; No
18. **If there was a break of more than 1 month, how did this affect your child's condition?** [Open-ended] ______
19. **What is the least amount of money you have spent for a month's worth of cannabis product?** [Free-text] ______ ($ Dollars)
20. **What is the most amount of money you have spent for a month's worth of cannabis product?** [Free-text] ______ ($ Dollars)
21. **Does the cost of the cannabis product place a significant strain on your finances?** [Multiple choice] Yes, always; Maybe sometimes; No

**CANNABIS PRODUCT INFORMATION (for each cannabis product sampled)**

1. **Current or past use of cannabis product:** Currently using; Previously used
2. **How long have you been using this cannabis product for?** [Free-text] ______ (Months)
3. **How did you obtain this cannabis product?** [Please tick any that apply] I grow or make my own; From friends or family; From a dealer of recreational cannabis products; From a medicinal cannabis product supplier; From a club or co-operative type outlet; Australian online supplier; Overseas online supplier; Overseas other source; Compassionate Access Scheme; Other
4. **If selected 'Other', please specify:** [Open-ended] ______
5. **Please describe the type of cannabis product:** [Please tick any that apply] Oil-based tincture; Alcohol-based tincture; Tablets/capsules; Solid or paste-like substance (e.g., hash, hemp seeds); Plant matter; Other
6. **If selected 'Other', please specify:** [Open-ended] ______
7. **What method did you or your supplier use to make the cannabis-based product?** [Please tick one] Alcohol-based cold extraction (liquid); Oil-based low heat extraction (liquid); Sift method (hash/kief); Sift method with dry ice (hash/kief); Raw form for smoking/vaporizing; I do not know; Other
8. **If selected 'Other', what is the method?** [Free Text] __________
9. **What information do you have regarding the composition of the cannabis product?** [Multiple choice] Written analysis from supplier; Internet; Word of mouth; No information; Other
10. **If selected 'Other', please specify:** [Free Text] __________
11. **What are the active ingredients of the cannabis product?** [Please tick any that apply] THC; THCA; THCV; THCVA; CBD; CBDA; CBC; CBDV; CBGA; CBG; CBN; I do not know
12. **Is there a recommended dose for this particular cannabis product?** Yes; No
13. **In what form is the recommended dose typically given?** [Multiple choice] Volume of drug solution (mL); Volume of drug solution (no. of drops); Weight of drug (mg/kg); Dosage form (tablet, capsule); Other quantity
14. **How many mL/ mg per kg/ drops /capsule or tablets or other dose form do you administer per day?** [Free Text] __________ (Dose per day)
15. **How many times a day is the dose administered?** [Free Text] __________ (number)
16. **At what times of the day do you administer the dose?** [Please tick any that apply] Morning; Afternoon; At night/before bed; As needed; No specific time of the day; Other
17. **If chosen "Other", in what other circumstances do you administer the cannabis product to your child with epilepsy?** [Free Text] _________
18. **What made you select this dose?** [Please tick any that apply] Trial and error based on impact on seizures; Trial and error based on side effects; Recommendation by supplier; Recommendation by word of mouth; Recommendation by health provider; Cost; Other
19. **If selected 'Other', please specify:** [Free Text] ______
20. **How long have you been using this particular dose?** [Free Text] _______ (In Months, Weeks or Days)
21. **At what stage of up-titrating to the maximum dose of the cannabis product are you?** [Multiple choice] Still at the starting dose; Less than half way to maximum dose; Half way to maximum dose; More than half way to maximum dose; At maximum dose; Not applicable
22. **How do you administer the product?** [Please tick any that apply] Shake the bottle first; Under the tongue (on its own); On the tongue (on its own); In the mouth (on its own); Mixed in with food, drink, or other medication; Smoked; Vaporised; Via the nose; Suppository; Topical (e.g. skin patch); Peg feed; Other
23. **If "Other", please specify:** [Free Text] _______
24. **Do you ever vary the dose?** Yes; No
25. **If you do vary the dose, what factors influence this?** [Please tick any that apply] Titrating to maximum dose; Unfavourable side effects; Availability due to cost/supply; To adjust for child's development (i.e. height and weight); Perceived effectiveness on seizure activity; Other
26. **If selected 'Other', please specify:** [Free Text] ______
27. **Do you think that the composition of the product is the same from batch to batch?** [Multiple choice] Always varies; Somewhat varies; Not sure; Usually the same; Always the same
28. **How do you normally store the cannabis product?** [Please tick any that apply] In an airtight container; Plastic container; Glass container; In a dark/amber coloured container; Other
29. **If selected 'Other', please specify:** [Free Text] ______
30. **Where do you normally store the cannabis product?** [Please tick any that apply] At room temperature (e.g., on the kitchen bench/ cupboard); In the fridge; In the freezer; Away from direct sunlight (e.g. inside cupboard/fridge, or wrapped in tin foil); Other
31. **If selected 'Other', please specify:** [Free Text] ______
32. **Does the cannabis product have an expiry date that you aware of?** Yes; No
33. **How long does it typically last for in terms of its "use by date"?** [Free Text] ______ (Months)
34. **How many antiepileptic drugs was your child on PRIOR to starting this particular cannabis product?** [Free Text] ______ (Number)

**CANNABIS PRODUCT PERCEIVED EFFICACY (Data collected for each cannabis extract sample provided)**

1. **Since starting to use the cannabis product, how would you globally report it's overall effectiveness in managing your child's epilepsy?** [Multiple choice] Very much worse; Much worse; Minimally worse; No change; Minimally improved; Much improved; Very much improved
2. **What was the percentage (%) change in average seizure frequency AFTER commencing the cannabis product?** [Free-text] ________ (Please write down a percentage figure +/- between 0 - 100%
3. **What was the longest seizure-free time period AFTER commencing the cannabis product?** [Free Text] ______ (Days)
4. **How many antiepileptic drugs is your child with epilepsy using in addition to the cannabis product?** [Free Text] ______ (Numerical)
5. **What are the names of the AEDs currently used?** [Please tick any that apply] Tegretol (Carbamazepine/ Carbatrol/ Mazepine); Zarontin (Ethosuximide); Felbamate (Felbatol); Keppra (Levetiracetam); Lamictal (Lamotrigine); Epilum (Sodium Valproate); Clobazam (Frisium); Topamax (Topiramate); Lacosamide (VIMPAT); Lyrica (Pregabalin); Gabitril (Tiagabine); Fycompa (Perampanel); Dilantin (Phenytoin/ Tremytoine); Valproate (Volproic acid/ Depakene/ Depakote); Zonegran (Zonisamide); Phenobarbitol (Phenobarb/ Phenobarbital Sodium); Clonazepam (Rivotril/ Clonpam, Clonazepam-R); Diazepam (Valium/ Diastat/ Diazemuls/ Dipam); Gabapentin (Neurontin); Fosphenytoin (Cerebyx); Lorazepam (Ativan/ Loraz); Methsuximide (Celontin); Oxcarbazepine (Trileptal/ Oxtellar XR); Nitrazepam (Mogadon/ Nitrazedon); Paraldehyde; Primidone; Rufinamide (Banzel); Vigabatrin (Sabril); Stiripentol; Other.
6. **If Other, what is the name of the AED(s)?** [Free Text] ______
7. **Have you weaned off or reduced any of your child's antiepileptic medications after starting any of the cannabis products?** [Multiple choice] Yes, all; Some medication but not all; No, none
8. **Have you noticed any harmful effects (side effects) since using the cannabis product? Please list.** [Open-Ended] __________
9. **If you listed any harmful effects, how confident are you that these are due to the cannabis product?** [Multiple choice] Really not confident; Not confident; Unsure; Confident; Really confident; I did not notice any harmful side effects
10. **Have you noticed any beneficial effects since using the cannabis product? Please list.** [Open-Ended] __________
11. **If you listed any beneficial effects, how confident are you that these are due to the cannabis product?** [Multiple choice] Really not confident; Not confident; Unsure; Confident; Really confident; I did not notice any beneficial effects
12. **Have you noticed any changes in their thinking skills and memory since using the cannabis product?** [Multiple choice] Worse; No change; Better; Not applicable (e.g. too young) Attention/ concentration; Memory; Speed of thinking; Language- Ability to express themselves; Language- Ability to understand what others are saying to them; Visual and motor skills; Ability to plan and organize his/herself; Ability to understand and solve problems; Ability to control his/her impulses; Ability to maintain focus despite distractions and shift focus when appropriate
13. **How would you report the overall effect on your child’s PHYSICAL activity since using the cannabis product?** [Multiple choice] Much worse; Somewhat worse; Stayed the same; Somewhat better; Much better
14. **How would you report the overall effect on your child's SLEEP since using the cannabis product?**[Multiple choice] Much worse; Somewhat worse; Stayed the same; Somewhat better; Much better
15. **How would you report the overall effect on your child's EMOTIONAL WELL-BEING since using the cannabis product?** [Multiple choice] Much worse; Somewhat worse; Stayed the same; Somewhat better; Much better
16. **How would you report the overall effect on your child's SOCIAL ACTIVITIES since using the cannabis product?** [Multiple choice] Much worse; Somewhat worse; Stayed the same; Somewhat better; Much better
17. **How would you report the overall effect on your child's BEHAVIOUR inside and outside the home since using the cannabis product?** [Multiple choice] Much worse; Somewhat worse; Stayed the same; Somewhat better; Much better

**CANNABIS PRODUCT EXPERIENCES**

1. **Please indicate the extent to which each of the following concern you with regards to the use of cannabis product, whether or not you are using cannabis products:** [Multiple choice] Not at all; A little; Not sure; Quite a bit; A great deal = Illegal status of cannabis & risk of criminal changes; Potential short-term health effects of cannabis use; Potential long-term health effects of cannabis use; Potential interactions with antiepileptic medication; Composition of the cannabis product (i.e. heavy metals, fungal or bacterial infections, toxins or pollutants); Risk notification to child protection by health providers or hospital staff; Risk notification to child protection by school / others; Cost of cannabis products; Stigma from health providers; Stigma amongst family / friends; Other
2. **Has anyone in the household ever used cannabis for any reason (e.g. medical or recreational)?** Yes; No
3. **Has anyone in the household used cannabis in the past 12 months**? Yes; No
4. **Have you told any health service providers about the use of cannabis products for your child's epilepsy?** Yes; No
5. **If not, please explain why not?** [Open-Ended] __________
6. **Do you feel that you and your family receive an adequate level of support regarding your decision to use cannabis products for your child's epilepsy?** [Multiple choice] Yes - with most of my health care providers; Yes - but only with a small number of health care providers; No - not with any of my health care providers
7. **Please indicate which type of health service provider and their response or reaction in the table below** [Multiple choice] Very unsupportive; Unsupportive; Uncertain; Generally supportive; Very supportive; They do not know = Neurologist (general or specialising in children or epilepsy); Paediatrician; General Practitioner (GP); Nurse; Other health professional
8. **What are your family and friends' general attitude to your use of cannabis products for your child's epilepsy?** [Multiple choice] Very unsupportive; Unsupportive; Uncertain; Generally supportive; Very supportive; They do not know
9. **How often have you engaged with health services for your child's epilepsy since starting the last treatment?** [Multiple choice] Much more often; More often; About the same; Less often; Much less often
10. **Please estimate the average number of times per month you have engaged with health services in relation to your child's epilepsy in the past year?** [Free-Text] __________
11. **Have you and your family experienced any of the following in the context of using cannabis products for your child?** [Please tick any that apply] Police involvement, arrests and/or charges; Child protection involvement; Cannabis products confiscated; Cannabis products not allowed to be used during hospital admissions; Difficulties with interstate or international travel whilst in possession of cannabis products

**FUTURE USE OF CANNABIS PRODUCTS**

1. **What should be the legal status of cannabis use in Australia?**[Multiple choice] Cannabis use should be legal for all purposes (recreational or medical); Cannabis use should be legal for medical purposes only; Cannabis use should remain illegal for all purposes; I'm not sure; No opinion
2. **If medical cannabis were legally available in Australia, what form of cannabis use would you most likely prefer if it were to help treat your and your family member's medical illness?** [Please tick any that apply] Oral use - Tablets/capsules (swallowed); Oral use - Chewable tablet; Oral use - Liquid (e.g. oil); Mouth spray; Edible (e.g., butter/resin, tea infusion); Smoked – Joint; Smoked - Water pipe ("bong"); Vaporiser; Via the nose - intranasal spray; Via the nose - Inhalant (e.g. like "Vick's vapor"); Suppository; Skin patches; I do not care as long as it is effective; All of them; Other
3. **If selected 'Other', please specify:** [Free Text] _____
4. **If medical cannabis were legally available in Australia, how would you most prefer to access medical cannabis products?** [Please tick one] Grow or make your own; Trade / buy from a friend; From a special licensed cannabis dispensary; From a pharmacy like other medication; From a supplier; I do not care as long as there is regular supply; No opinion; Other
5. **Please indicate the degree to which you agree or disagree with each of the following statements regarding medical cannabis.** [Multiple choice] Strongly disagree; Disagree; Neither agree or disagree; Agree; Strongly agree; No opinion

**Supplementary Methods S2**

**Sample analysis**

*Chemicals and reagents*

Acetonitrile, formic acid, ethanol, methanol, and methyl-*tert­-*butyl ether were obtained from Fisher Scientific (Melbourne, VIC, Australia). Phytocannabinoid reference standards and deuterated internal standards of cannabidiol (CBD), cannabidiolic acid (CBDA), cannabidivarin (CBDV), cannabidivaric acid (CBDVA), ∆^9^-tetrahydrocannabidiol (∆^9^-THC), ∆^9^-THC-*d*_3_, tetrahydrocannabidiolic acid (THCA), tetrahydrocannabivarin (THCV), cannabichromene (CBC), cannabigerol (CBG), cannabigerolic Acid (CBGA), cannabinol (CBN), 11-nor-carboxy-THC, (THC-COOH), THC-COOH-*d*_3,_ and 11-hydroxy-THC (11-OH-THC), were purchased from Cerilliant (Round Rock, TX, USA). Red abalone β-glucuronidase was purchased from PM separations (Capalaba, QLD, Australia), and creatinine reference standard from Sigma-Aldrich (Castle Hill, NSW, Australia). Terpenoid reference standards of (±)-α-pinene, camphene, (-)-β-pinene, β-myrcene, 3-carene, α –terpinene, p-cymene, D-limonene, 1,8-cineol, ocimene (mix of α and β ocimenes), γ-terpinene, terpinolene, β-linalool, (-)-isopulegol, geraniol, β-caryphyllene, humulene, nerolidol (mix of cis and trans isomers), (-)-caryophyllene oxide, (-)-guaiol, and (-)-α-bisabolol were purchased from Restek (Bellefonte, PA, USA). Lauryl acetate was purchased from Sigma-Aldrich. All chemicals and solvents were at least ACS or high-performance liquid chromatography grade, respectively.

*Phytocannabinoid extraction from oils and pastes*

Protocol for oils/pastes was adapted from DeBacker et al. (2009). Oils/pastes were aliquoted and weighed in quadruplet into glass 16 x 100 mm glass tubes. Ethanol was added to make a 10 mg/mL solution per the weight of the oil unless the sample was scarce then a 1 mg/mL solution was made. 10µL of diazepam (1 mg/mL) was used an internal standard. The tubes were capped and spun on a rotary mixer for 20 min. Samples were then sonicated using an ultrasonic water bath for 15 min at 18 °C. Samples were then winterized in the -80 ⁰C freezer for 30 min. Test tubes were then centrifuged in a Sigma 2-16PK centrifuge fixed with a swing out rotor at 5,000 x *g* for 10 minutes at -4 °C. 1 mL of each sample was then transferred into a new 13 x 100 mm glass test tube.

*Phytocannabinoid extraction from urine*

Phytocannabinoid urinalysis was performed as reported previously (Kevin et al., 2017) with minor modification. 0.5 mL of urine samples were aliquoted in triplicate into 12 x 75 mm glass tubes and spiked with a mixture of phytocannabinoid internal standards (THC-*d*_3_ and THC-COOH-*d*_3_) in methanol. Calibrator and quality control samples of known phytocannabinoid concentration were prepared by addition of reference standards to cannabinoid-free urine and treated identically to participant samples. Urinary hydrolysis was performed by adding 0.5 mL of 0.2 M sodium acetate pH 5 buffer to each sample, followed by 12.5 µL red abalone β-glucuronidase. Tubes were capped and incubated at 37 °C for 15 h.

Phytocannabinoids were extracted from urine using supported liquid extraction. 1 mL of sample solution was absorbed on Biotage Isolute SLE+ columns (Rydalmere, NSW, Australia). Analytes were eluted with 5 mL methyl-*tert­-*butyl ether into clean 12 x 75 mm glass tubes. Samples were evaporated to dryness under a stream of nitrogen, reconstituted in 100 µL of 60:40 0.1% formic acid and acetonitrile, and transferred to amber vials for immediate analysis via LC-MS/MS.

*Creatinine extraction from urine*

10 µL of urine samples were aliquoted in duplicate into 12 x 75 mm glass tubes. 490 µL chilled 0.1% formic acid in acetonitrile was added, and each sample was centrifuged at 5,000 x *g* for 10 min at 4 °C. 25 uL of supernatant was transferred to amber vials, and 225 uL of 0.1% formic acid and 250 uL of 0.1% formic acid in acetonitrile were added to each vial, giving a total dilution factor of 1000 x relative to initial sample concentration. A standard curve was generated ranging from 50 to 5000 ug/mL via addition of creatinine reference standard to water and extracted as specified above. Samples were analysed immediately via LC-MS/MS.

*Terpenoid extraction from oils and pastes*

10 mg of each oil or paste was aliquoted in duplicate into 12 x 75 mm glass tubes, and spiked with 10 µL of a 1 mg/mL solution of lauryl acetate (internal standard) in water. 2 mL ethanol was added to each tube. The tubes were capped, briefly vortexed, and sonicated for 15 minutes at 18 ⁰C. Samples were then centrifuged at 5000 x *g* for 10 minutes at -4 ⁰C. 1 mL of supernatant was transferred to amber vials for immediate analysis via gas chromatography-mass spectrometry (GC-MS).

*LC-MS/MS analysis of phytocannabinoids and creatinine*

Quantification of phytocannabinoids extracted from oils, pastes, or urine samples was performed using a Shimadzu Nexera® ultra-high-performance liquid chromatograph (Shimadzu Corp., Kyoto, Japan) coupled to a Shimadzu LCMS-8030 triple quadrupole mass spectrometer. 20µL injections of each sample, kept in a 4 °C autosampler, were chromatographically separated using a Zorbax C8 guard column in conjunction with a Zorbax XDB-C18 reverse-phased analytical column (100 x 2.1 mm i.d., particle size 3.5 µm; Phenomenex, Lane Cove, NSW, Austalia). This was performed via gradient elution with 0.1% formic acid in water and acetonitrile at a flow rate of 0.3 mL/min. The mass spectrometer was operated in positive electrospray ionization mode with multiple reaction monitoring to identify and quantify analytes. Quantification of creatinine was performed as specified above, except that isocratic 0.1% formic acid in 50% acetonitrile was used as the mobile phase.

*GC-MS analysis of terpenoids*

Terpenoid quantification was performed using a Shimadzu GC-2010 Plus GC system coupled to a Shimadzu QP-2010 MS. The GC-MS system was also equipped with a AOC-20i/s autosampler (Shimadzu Corp.) and RTX-5MS column (30 m length, 0.25 mm i.d., film thickness x 0.25 µm; Restek). The injection port was kept at 250 °C and 1 µL of sample was injected with a split ratio of 10:1 and a helium carrier gas column flow rate of 1 mL/min. The column oven temperature was held at 40 °C for 2 min, before increasing to 150 °C at a rate of 10 °C /min, where it was held for 12 min until a final 25°C/min ramp to 310 °C and held for 10 min for a total run time of 41.7 min. Analytes were detected using electron impact ionization (70 eV) in scan mode from *m/z* 50 – 400, with the ion source held at 200 °C. Sample analyte identity was confirmed via comparison to reference standards (in terms of retention time and mass spectra) and to the NIST EI mass spectral library (version 11). Analytes were quantified using the most abundant ion.

**Supplementary Methods S3**

**Thematic Analysis Report**

**Interview Question:** *Why did you decide to start using cannabis products for your child's epilepsy?*

| **Theme** | | **Examples of participant’s response at interview** |
| --- | --- | --- |
| Uncontrolled seizures & concerns for child’s well-being | “Nothing else was working for him; his concentration is getting worse…”  “No drug out there works for Dravet Syndrome…we were concerned about the risk of SUDEP…”  “We are desperate – trying everything; failed so many drugs”  “Her physical health was deteriorating…” | |
| Intolerable antiepileptic drug (AED) side-effects | “…very poor quality of life (from AED side-effects)”  “Clobazam was making her sedated/putting her out…”  “increasing side-effects with AEDs - cognitively stuffing him up…”  “Last medication she was on she developed stomach ulcers so had to stop…” | |
| Success stories (media) and word of mouth | “…heard success story of Charlotte Figi…”  “Parents of Dravet kids in the US inspired me to do it…”  “Neighbour recommended after seeing story on 60 minutes - she was using cannabis for terminal lung cancer…” | |
| Personal research into cannabis extracts for epilepsy | “Have looked at the clinical trials and research and thought that (we) needed to try something else…”  “…starting reading the research online (scientific articles not just blogs)…” | |
| To find natural alternative to pharmaceutical drugs | “(/we) like that it is a natural product, not another pharmaceutical…”  “My parents had been researching alternatives to drugs…” | |
| To manage health conditions in addition to epilepsy | “…help with wellbeing, autism, sleep and mood…”  “…wanted to use it for (child’s) autism too.” | |
| To try as a rescue medication | “…wanted to try it as a rescue drug. Midazolam only works after 20 minutes… want to avoid brain damage.” | |

**Interview Question:** *Have you previously trialed cannabis products for your child's epilepsy and stopped? If yes, why did you stop?*

| **Theme** | | **Examples of participant’s response at interview** |
| --- | --- | --- |
| Cannabis extract was ineffective at reducing seizures | “Worked to control seizures and improve functioning initially but dropped off in effectiveness after a while (honeymoon period)…”  “No change in seizure frequency.” | |
| Parent noticed side-effects after starting cannabis extracts | “…noticed child was lethargic…”  “We only used it for about three weeks…he was looking wasted, poor sleep and increased behavioural problems…” | |
| Problems with supply and/or access | “  “We tried three different suppliers and with each, something happened, their stock got seized, they got arrested or shut down by the police.”  “Supplier was unable to obtain any more supplies so we had to stop using…” | |
| Parent unsure how to use cannabis extracts | “We were not confident in what we were doing…” | |
| Concerned over quality of composition | “(We were) concerned over the ingredients...” | |
| Child too young / too difficult to administer oil | “Waiting until he gets older so he can safely swallow…” | |
| Reported to child protection and the police | “We had a visit from child safety and the police. We think they received information from (child’s) doctor…” | |
| Parent noticed possible drug interactions | “Her condition relies on treatment with benzos, which we observed to interact with cannabis, leading to ineffectiveness.” | |

**Interview Question:** *Why have you not tried cannabis products for your child's epilepsy to date?*

| **Theme** | | **Examples of participant’s response at interview** |
| --- | --- | --- |
| Fear of legal consequences and risk of child protection | “Too risky in (current) legal climate…concerned about child protection”  “…have seen horror stories in the media and am afraid daughter will be taken by DOCs…” | |
| Unsure how to access it | “We would love to use and trial cannabis but don’t know how to get access…”  “We looked at options in the USA. Interested in medicinal cannabis but having difficulty with accessing it here.” | |
| Concerned over safety risks and composition | “Safety issues…”  “Worried about the composition of illegal products…” | |
| I don’t know enough about it (dosage) | “…dosage issues…I’d be worried about dosing”  “Lack understanding (about) medicinal cannabis.” | |
| Need for medical supervision | “I don’t feel qualified to make treatment decisions, leave that to the doctor…”    “No assistance or support from health providers…” | |
| Health provider advised against it | “…neurologist advised (that) there’s no current need to try cannabis…” | |
| Seizures currently well-controlled | “Medication’s effective and not worth the risk…”  “Have not been desperate enough to take the risk…(child’s) seizures ok at the moment…” | |
| Lacks evidence for use in a specific type of epilepsy | “…concerned about lack of research…did own research and found no evidence for benefits for (my child’s) specific condition.” | |

**Interview Question:** *Have you noticed any beneficial effects since using the cannabis product? Please List.*

| **Theme** | | **Examples of participant’s response at interview** |
| --- | --- | --- |
| Improved cognition | Fs  “…concentrates better when we read to him. He seems more focused…”  “Her school work improved. Better academic results and seems to have a clearer head.”  “Developmentally progressed, eating more, more alert…” | |
| Emotional well-being | “Seems more present, happier, less shy…”  “She sings all the time and seems more joyful…” | |
| Language skills | “They (child) are talking now and show a better understanding of language.”  “…speech improved drastically.” | |
| Social activity | ”Seems less autistic…”  “Trying to talk more …. and better eye contact.”  “Her personality comes through more and we see her sense of humour more often” | |
| Physical activity | “More active…”  “Less ataxic…she can feed herself, dress herself now.”  “Muscle control and balance improved.” | |
| Sleep | “Better night time sleep. More energy and no sleeps during the day.”  “Wakes up happy and seems better rested.” | |
| Behaviour | “Helped improve behaviour. She has less rages.” | |

**Interview Question:** *Have you noticed any harmful effects (side-effects) since using the cannabis product? Please List.*

| **Theme** | | **Examples of participant response at interview** |  |
| --- | --- | --- | --- |
| Worsening of pre-existing behaviours | C “For the first two weeks she had no behaviours. Then it just went back to severe behaviours, worse than before.”  “Thought cannabis interacted with his valium, which made his behaviours worse.” | | |
| Drowsiness/lethargy | “Very minimal drowsiness in the morning.”  “Sleepy” | | |
| Gastrointestinal upset | “Tummy issues…”  “Nausea for five weeks before we started to dilute the cannabis oil and then he had less symptoms.” | | |
| Possible intoxication | “Made him look stoned…”  “Laughing (uncontrollably)…” | | |
| Changes to appetite or weight | “He wants to eat but as soon as he starts he loses his appetite.”  “In its undiluted form it made him gain a lot of weight…” | | |
| Possible increase in seizures | “Slight increase in tonic/clonic seizures…”  “Increase in cluster seizures from the oil. Particularly nocturnal.” | | |
| Temporary changes in sleep and mood | “Increase in product resulted in increase in anger/rage.”  “Some sleep issues and increased restlessness…” | | |

| **Epilepsy etiology** | **Epilepsy diagnosis** | **Total** |
| --- | --- | --- |
| **Genetic** |  |  |
|  | Dravet syndrome (All SCN1A) | 6 (9.2%) |
|  | Epileptic encephalopathy (DNM1 mutation) | 2 (3.1%) |
|  | Myoclonic astatic epilepsy (presumed genetic) | 2 (3.1%) |
|  | Continuous spike-and-wave during sleep epilepsy (SCN9A mutation & MCPH1 deletion) | 1 (1.5%) |
|  | Miller-Dieker syndrome (17p13.3 microdeletion) | 1 (1.5%) |
|  | Epileptic encephalopathy (PCDH19 mutation) | 1 (1.5%) |
|  | Tuberous sclerosis complex (TSC2 mutation) | 1 (1.5%) |
|  | Retts syndrome (C.316C>T mutation) | 1 (1.5%) |
|  | Epileptic encephalopathy (KCNQ2 mutation) | 1 (1.5%) |
|  | Uncharacterized epilepsy (Chromosome 16 triplicate) | 1 (1.5%) |
|  | Uncharacterized epilepsy (SCN2A mutation) | 1 (1.5%) |
|  | Uncharacterized epilespy (Chromosomal microdeletion 15, 17, X) | 1 (1.5%) |
|  | Uncharacterized epilespy (presumed genetic) | 1 (1.5%) |
|  | Photosensitive epilepsy | 1 (1.5%) |
|  | Lennox Gastaut syndrome | 1 (1.5%) |
| **Unknown** |  |  |
|  | I do not know | 27 (42%) |
|  | Epileptic encephalopathy with continuous spike-and-wave during sleep | 2 (3.1%) |
|  | Childhood absence epilepsy | 2 (3.1%) |
|  | West Syndrome | 1 (1.5%) |
|  | Aicardi syndrome | 1 (1.5%) |
|  | Cerebral palsy | 1 (1.5%) |
|  | Dravet Syndrome (suspected) | 1 (1.5%) |
|  | Febrile infection-related epilepsy syndrome | 1 (1.5%) |
|  | Landau-Kleffner syndrome | 1 (1.5%) |
|  | Myoclonic Rolandic childhood epilepsy | 1 (1.5%) |
|  | Occipital lobe epilepsy | 1 (1.5%) |
|  | Childhood absence epilepsy (atypical) | 1 (1.5%) |
|  | Juvenile myoclonic epilepsy | 1 (1.5%) |
|  | Myoclonic astatic epilepsy | 1 (1.5%) |
|  | Nocturnal frontal lobe epilepsy | 1 (1.5%) |
|  | ‘Lennox Gastaut’-like syndrome | 1 (1.5%) |
|  |  |  |
| **Structural** |  |  |
|  | Acquired brain injury | 2 (3.1%) |
|  |  |  |
| **Metabolic** |  |  |
|  | Pyridoxine-dependent epilepsy | 1 (1.5%) |
|  |  |  |
|  | **Total** | **65** |

**Supplementary Table S1.** List of diagnoses as reported by families of children with epilepsy (N=65)

**Supplementary Table S2.** Child Behaviour Checklist (CBCL) mean T-scores and percentage of children at risk for behaviour problem for children aged 1.5 – 5 years and 6 – 16 years by cannabis extract use history.

| **Age 1.5 – 5 years** |  |  |  |
| --- | --- | --- | --- |
| **Measure** | **Currently or previously using cannabis extracts (n=12)** | **Never used cannabis extracts (n=9)** | **Total (n=21)** |
|  | Mean (SD) [% at risk] | Mean (SD) [% at risk] | Mean (SD) [% at risk] |
| Total problems | 52.6 (8.5) [ - ] | 54.3 (15.6) [33%] | 53.3 (11.2) [14%] |
| Internalising | 56.7 (10.8) [8%] | 58.3 (10.5) [11%] | 57.4 (10.4) [10%] |
| Externalising | 54.7 (9.8) [8%] | 52.8 (13.9) [11%] | 53.9 (11.4) [10%] |
|  |  |  |  |
| **Subscales** |  |  |  |
| Emotionally reactive | 57.6 (9.5) [17%] | 57.2 (10.9) [11%] | 57.4 (9.9) [14%] |
| Anxious/Depressed | 53.9 (6.1) [ - ] | 51.4 (0.7) [ - ] | 52.9 (5.0) [ - ] |
| Somatic complaints | 55.8 (6.1) [ - ] | 62.3 (9.6) [22%] | 58.6 (8.3) [10%] |
| Withdrawn | 65.9 (11.7) [33%] | 62.2 (13.7) [22%] | 64.3 (12.4) [29%] |
| Sleep problems | 57.6 (9.7) [8%] | 53.4 (5.3) [ - ] | 55.8 (8.2) [5%] |
| Attention problems | 63.8 (6.8) [8%] | 58.6 (7.6) [ - ] | 61.6 (7.5) [5%] |
| Aggressive behaviour | 54.2 (5.8) [8%] | 55.8 (9.9) [11%] | 54.9 (7.7) [10%] |
| **Age 6 - 16 years** |  |  |  |
| **Measure** | **Currently or previously using cannabis extracts (n=22)** | **Never used cannabis extracts (n=13)** | **Total (n=35)** |
|  | Mean (SD) [% at risk] | Mean (SD) [% at risk] | Mean (SD) [% at risk] |
| Total problems | 64.2 (10.3) [46%] | 68.4 (6.8) [54%] | 65.7 (9.3) [49%] |
| Internalising | 61.3 (10.9) [23%] | 67.4 (8.3) [46%] | 63.6 (10.4) [31%] |
| Externalising | 56.3 (12.8) [5%] | 61.5 (8.8) [15%] | 58.2 (11.7) [9%] |
|  |  |  |  |
| **Subscales** |  |  |  |
| Anxious/depressed | 59.5 (9.7) [18%] | 59.3 (9.5) [15%] | 59.4 (9.5) [17%] |
| Withdrawn/depressed | 60.4 (10.9) [23%] | 62.8 (8.3) [15%] | 61.3 (9.9) [20%] |
| Somatic complaints | 63.4 (11.0) [27%] | 72.6 (11.8) [62%] | 66.8 (12.0) [40%] |
| Social problems | 65.6 (8.5) [36%] | 71.8 (8.5) [46%] | 67.9 (8.9) [40%] |
| Thought problems | 65.6 (9.3) [32%] | 68.5 (6.7) [62%] | 66.7 (8.5) [43%] |
| Attention problems | 71.3 (14.8) [32%] | 76.7 (11.7) [62%] | 73.3 (13.8) [43%] |
| Disobedient behaviour | 56.5 (6.0) [5%] | 54.2 (13.5) [ - ] | 55.7 (9.4) [3%] |
| Aggressive behaviour | 61.0 (8.5) [18%] | 65 (12.8) [23%] | 62.6 (10.3) [20%] |

*Note*: T scores equal to or less than 50 represent few or no behavioural problems, whereas the highest score 100 indicates extensive behavioural problems. T scores ≥ 65 indicate child at or above the borderline clinical range (i.e. at risk).

**Supplementary Table S3.** TNO-AZL Preschool Children Quality of Life Questionnaire (1 – 5 years) and Quality of Life Childhood Epilepsy (QOLCE) (6 – 16 years) mean scale scores and standard deviation (SD) by cannabis extract use history.

| TNO-AZL Preschool Children Quality of Life (TAPQOL) (1 – 5 years) | | |
| --- | --- | --- |
| **Scale** | **Currently or previously using cannabis extracts (n=14)** | **Never used cannabis extracts (n=9)** |
|  | Mean (SD) | Mean (SD) |
| Sleep problems | 58.9 (21.5) | 62.1 (17.7) |
| Appetite | 64.3 (28.9) | 79.6 (14.5) |
| Lung problems | 83.3 (23.3) | 89.8 (18.1) |
| Stomach problems | 76.2 (25.3) | 78.7 (23.6) |
| Skin problems | 88.1 (12.5) | 93.5 (9.1) |
| Problem behaviour | 53.1 (28.5) | 67.5 (25.3) |
| Anxiety | 77.4 (26.6) | 72.2 (22) |
| Positive mood | 89.3 (21.3) | 96.2 (11.1) |
| Liveliness | 86.9 (21.9) | 77.8 (34.3) |
| Motor function | 62.1 (28.3) | 62.5 (32) |
| Social function | 58.3 (42.2) | 55.6 (37.3) |
| Communication | 55.4 (27.7) | 66 (24.5) |
| Quality of Life Childhood Epilepsy 55-item (QOLCE-55) (6 – 16 years) | | |
| **Scale** | **Currently or previously using cannabis extracts (n=22)** | **Never used cannabis extracts (n=15)** |
|  | Mean (SD) | Mean (SD) |
| Physical function | 26.4 (17.5) | 24.8 (17.9) |
| Social function | 39.3 (25.4) | 54.2 (16.0) |
| Cognitive function | 34.8 (30.2) | 32.9 (26.8) |
| Emotional well-being | 62.3 (15.6) | 29.4 (12.9) |
| Total quality of life | 40.8 (17.5) | 35.3 (10.2) |

Scores on the TAPQOL and QOLCE-55 are transformed to a score ranging from 0 to 100, with higher scores indicating better quality of life.

**
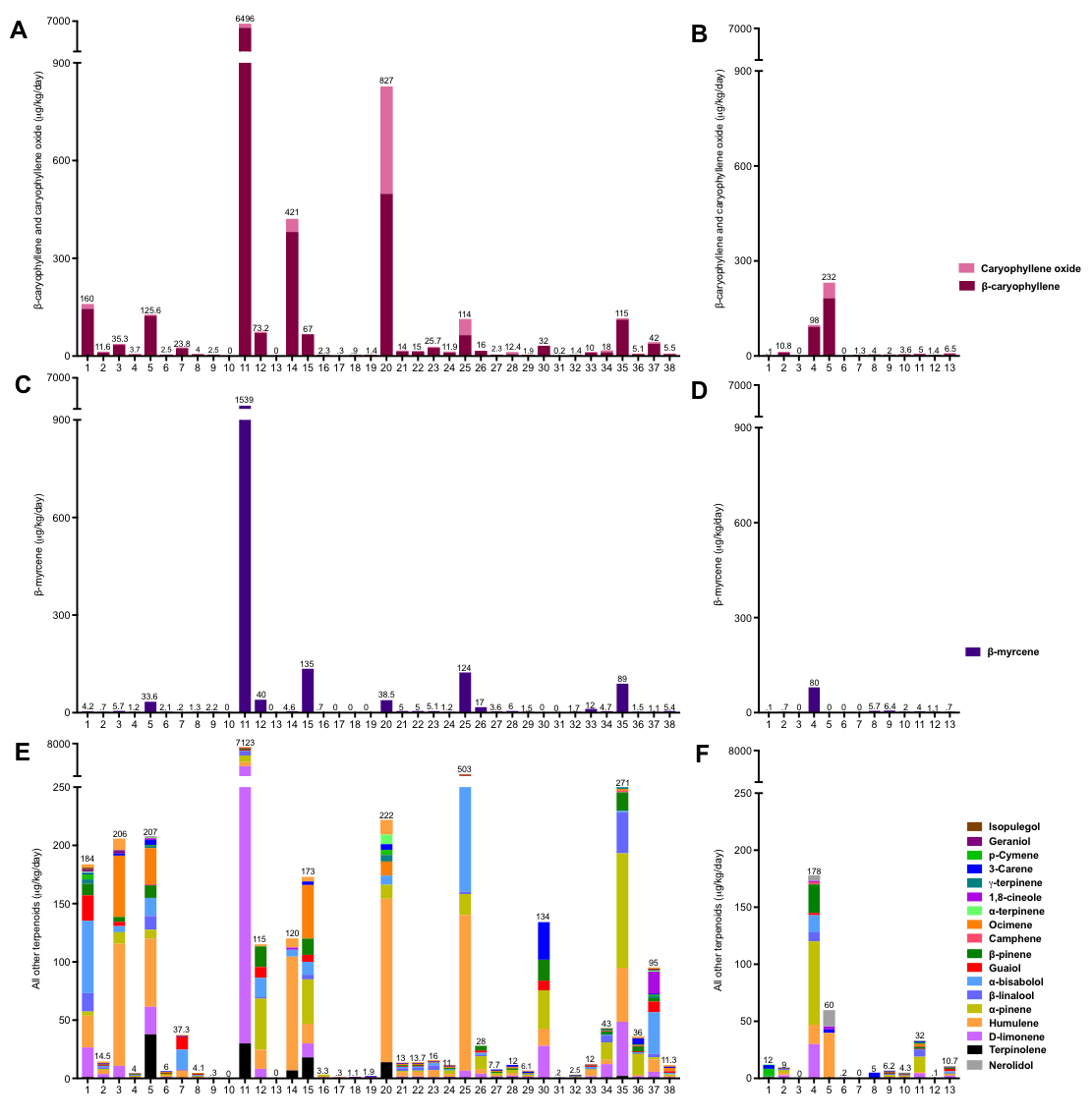
Supplementary Figure S1**. **Dose of terpenoids found in individual cannabis extract samples that were perceived “effective” or “ineffective” according to the family.** Dose of terpenoids in 51 cannabis extract samples collected from families using cannabis extracts to treat their child’s epilepsy. Samples perceived “effective” **(A, C, E)** and “ineffective” **(B, D, F)** are depicted in separate graphs. Dose of β-caryophyllene and its’ degradant, caryophyllene oxide **(A-B)**, β-myrcene **(C-D)**, and trace terpenoids **(E-F)** are depicted in micrograms (μg) per kilogram (kg) of child’s body weight per day. Note the scale of the Y-axis is different between the graphs. Values above each column indicate total terpenoid content for that graph (μg/kg/day).
